# Drug resistance and physiological roles of RND multidrug efflux pumps in *Salmonella enterica*, *Escherichia coli* and *Pseudomonas aeruginosa*

**DOI:** 10.1099/mic.0.001322

**Published:** 2023-06-15

**Authors:** Seiji Yamasaki, Martijn Zwama, Tomohiro Yoneda, Mitsuko Hayashi-Nishino, Kunihiko Nishino

**Affiliations:** ^1^​ SANKEN (The Institute of Scientific and Industrial Research), Osaka University, 8-1 Mihogaoka, Ibaraki, Osaka 567-0047, Japan; ^2^​ Graduate School of Pharmaceutical Sciences, Osaka University, 1-6 Yamadaoka, Suita, Osaka 565-0871, Japan; ^3^​ Institute for Advanced Co-Creation Studies, Osaka University, 1-1 Yamadaoka, Suita, Osaka 565-0871, Japan; ^4^​ Center for Infectious Disease Education and Research, 2-8 Yamadaoka, Osaka University, Suita, Osaka 565-0871, Japan

**Keywords:** drug efflux pumps, resistance–nodulation–division family, multidrug resistance, *Salmonella*, *Escherichia coli*, *Pseudomonas aeruginosa*, physiological function

## Abstract

Drug efflux pumps transport antimicrobial agents out of bacteria, thereby reducing the intracellular antimicrobial concentration, which is associated with intrinsic and acquired bacterial resistance to these antimicrobials. As genome analysis has advanced, many drug efflux pump genes have been detected in the genomes of bacterial species. In addition to drug resistance, these pumps are involved in various essential physiological functions, such as bacterial adaptation to hostile environments, toxin and metabolite efflux, biofilm formation and quorum sensing. In Gram-negative bacteria, efflux pumps in the resistance–nodulation–division (RND) superfamily play a clinically important role. In this review, we focus on Gram-negative bacteria, including *Salmonella enterica *, *Escherichia coli* and *Pseudomonas aeruginosa *, and discuss the role of RND efflux pumps in drug resistance and physiological functions.

## Introduction

The mechanisms by which bacteria acquire resistance to antimicrobial agents can be classified into four types: inactivation of antimicrobial agents by degradation or chemical modification; antimicrobial resistance (AMR) due to a change in the antimicrobial target, in which the site of antimicrobial action is mutated in the bacteria; drug resistance due to changes in membrane permeability; mechanisms that cause bacteria to become resistant by actively exporting antimicrobials and reducing their intracellular concentrations [1]. The first two mechanisms confer resistance to specific or the same class of antibiotics, while the latter two mechanisms increase resistance to a variety of structurally unrelated antibiotics. In many clinically isolated multidrug-resistant (MDR) bacteria, multiple mechanisms are present in the same strain, causing bacterial resistance to many drugs. Because a single multidrug efflux pump can export many structurally unrelated substrates, the elevated expression of even one pump can cause bacteria to become resistant to many compounds.

Bacterial drug efflux pumps can be classified into the following seven families based on their energy utilization and structural aspects: (1) the major facilitator superfamily (MFS); (2) the resistance–nodulation–division (RND) superfamily; (3) the small multidrug resistance (SMR) family; (4) the ATP-binding cassette (ABC) superfamily; (5) the multidrug and toxic compound extrusion (MATE) family; (6) the proteobacterial antimicrobial compound efflux (PACE) family; and (7) the *p*-aminobenzoyl-glutamate transporter (AbgT) family [2–6].

Drug efflux pumps, named because of their involvement in bacterial drug resistance, have been conserved across bacterial species since before humans used antimicrobial agents [7, 8]. These efflux pumps may have been necessary to counter competition among heterologous bacteria in the environment with antibiotic-producing microbes [9]. Additionally, drug efflux pumps play a fundamental role in survival in various natural environments, including host environments. In particular, RND-type efflux pumps are of interest because of their many functions: pH tolerance and resistance to toxic natural products, metal ions, bile acids, fatty acids and antimicrobial peptides produced by host cells. These RND-type pumps are coupled to an outer membrane protein (OMP), bridged together by periplasmic adaptor proteins (PAPs). Multidrug efflux pumps are also involved in biofilm formation. As 90 % of naturally occurring bacteria form biofilms, which are involved in causing clinically significant infections in host cells, including in humans, the impact of efflux pumps on biofilm formation and drug resistance is immense. Furthermore, several bacterial species perform intercellular communication through quorum sensing. Although quorum sensing signals (QSSs) accumulate in proportion to cell density, efflux pumps also play an essential role in regulating quorum sensing processes.

**Fig. 1.**
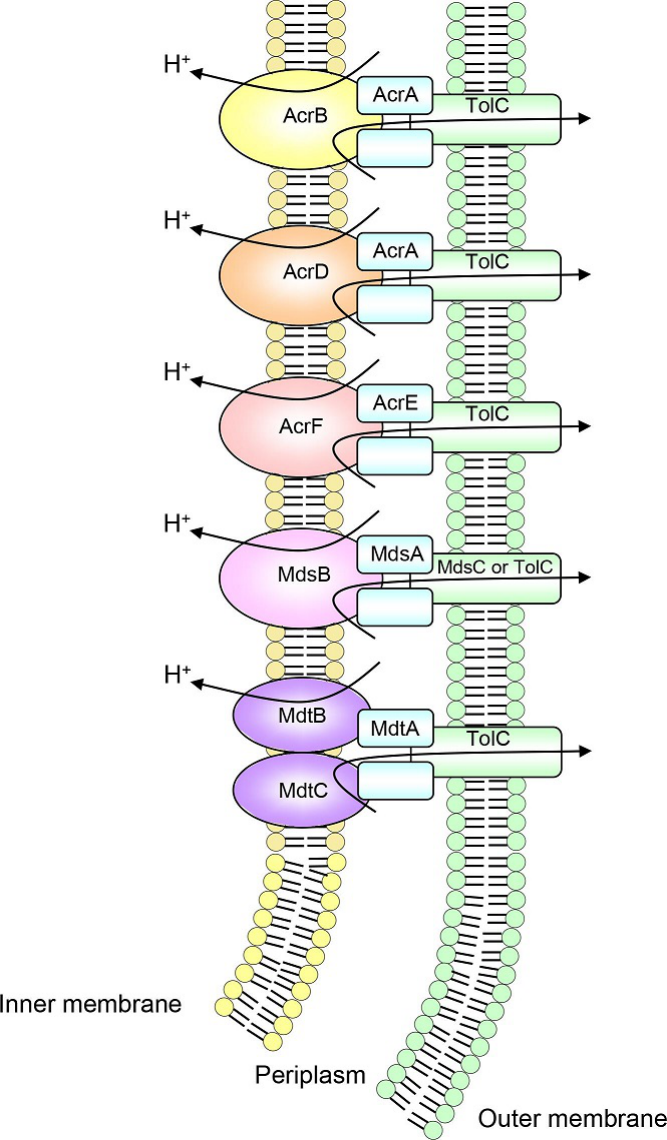
RND multidrug efflux pumps in *S. enterica *.

**Fig. 2.**
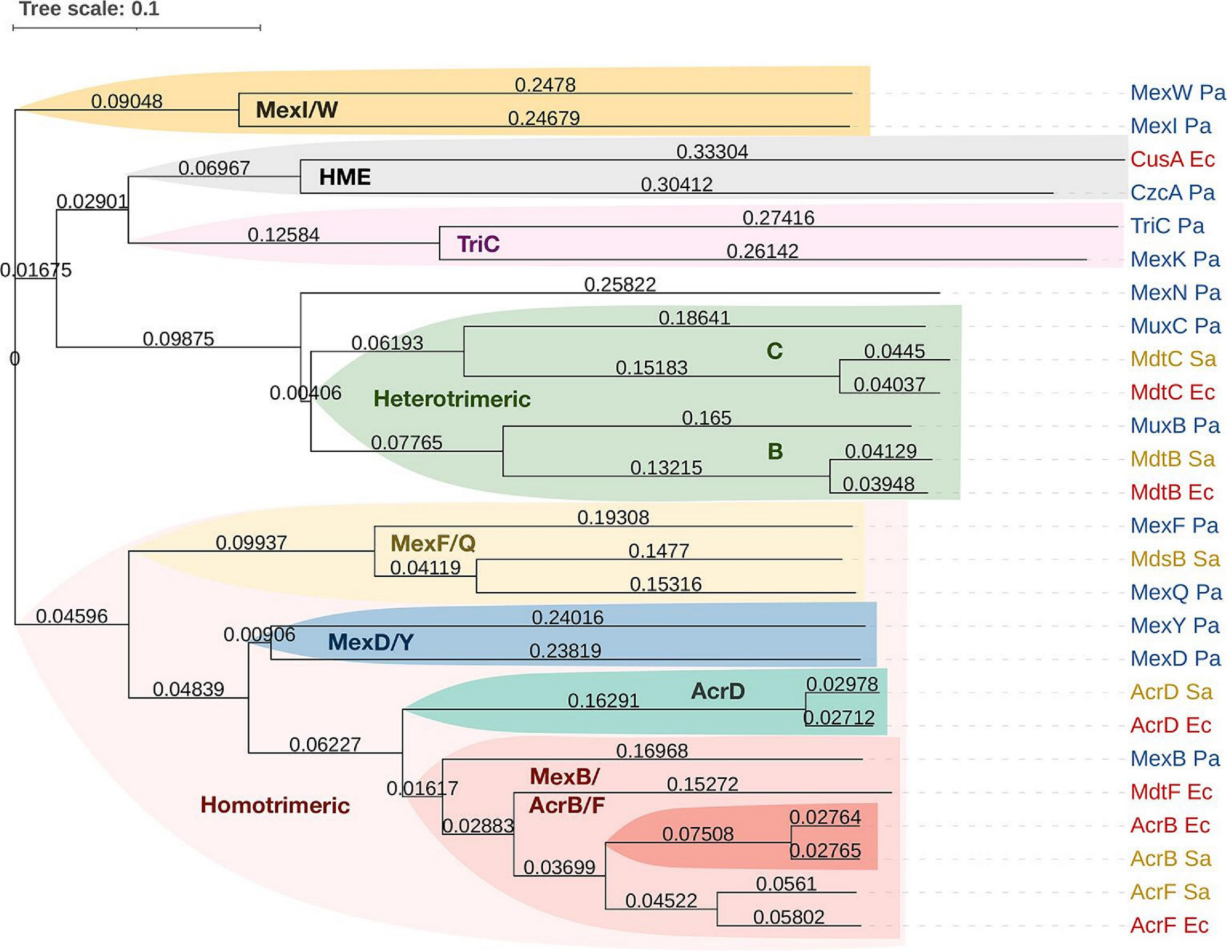
Phylogenetic tree of 26 RND-type efflux pumps. Phylogenetic clusters are highlighted in different colours. The three main clusters are the MexI/W-family, the TriC/HME and heterotrimeric-like pumps and the homotrimeric pumps. Multiple sequence alignment was performed using Kalign v3 [146], and the tree was made with iTol [147]. HME, heavy metal efflux subfamily; Pa, *P. aeruginosa *; Ec, *E. coli *; Sa, *Salmonella *. Colour protein names: blue, *P. aeruginosa *; red, *E. coli *; yellow, *Salmonella *.

This review summarizes the drug resistance and physiological roles of RND multidrug efflux pumps in *Salmonella enterica* (Fig. 1), *Escherichia coli* (Fig. 4) and *Pseudomonas aeruginosa* (Fig. 5). We focus on these three bacteria because they are clinically important in human coexistence and because the characteristics of their RND multidrug efflux pumps have been studied in more experiments than those of other species. A phylogenetic comparison between all 26 RND-type efflux pumps mentioned in this review can be seen in Fig. 2 and their sequences are in Table S1 (available in the online version of this article).

## The role of RND multidrug efflux pumps in *S. enterica*

*S. enterica* is an intracellular pathogen in animals and humans. This bacterium causes diseases ranging from gastroenteritis to life-threatening systemic infections. *S. enterica* contains at least 10 functional drug efflux pumps that belong to 4 (super) families: MFS (i.e. EmrAB, MdfA and SmvA), RND (i.e. AcrAB, AcrEF, AcrD, MdsAB and MdtABC), MATE (i.e. MdtK) and ABC (i.e. MacAB). Each efflux pump plays an important role not only in antibiotic efflux but also in physiological functions. Although the role of some efflux pumps has yet to be elucidated, we summarize the broad function of each RND pump in the following sections based on the recent literature (Fig. 1, Table 1). For coupling with the RND efflux pumps, the outer membrane component TolC is expressed from a separate operon in the genome, except for MdsABC, where MdsC is expressed from the same operon with MdsAB (Table S2).

**Table 1.** Drug resistance and physiological roles of RND multidrug efflux pumps in *S. enterica, E. coli* and *P. aeruginosa
*

Efflux pumps	Physiological functions	Substrates
*** S. enterica ***		
**AcrAB–TolC**	Cell adhesion [15, 24] Cell invasion [15, 16, 24, 26] Virulence [10, 16, 17, 26] Biofilm formation [21] Cell metabolism [32–34, 36]	ACR, ETBR, CIP, NAL, CHL, TET, NOV, FUS, ATM, CAZ, CTX, ERY, FQ, OXA, MIN, NOR, AMX, CRO, AMP, TRIC, cyclohexane, CLO, NAF, LVX, TMP, R6G, CV, BENZ, DOC, DXR, FAM, RIF, LZD, TRC, CAR, TPP, MB, SDS, TritonX-100, anti-histamine agent, plant alkaloids, anti-depressants, anti-psychotic drugs, anti-protozoal drugs, bile salts, fatty acids, steroid hormones [10, 16, 26, 32, 33, 148–154]
**Acr(A)D-TolC**	Cell adhesion [24] Cell invasion [24] Swarming motility [38] Biofilm formation [21] Fitness cost adjustment [38] Cell metabolism [38–40]	OXA, CLO, NAF, CAR, SB, ATM, SDS, NOV, Cu, Zn, sodium tungstate [37, 39, 40]
**AcrEF-TolC**	Cell adhesion [24] Cell invasion [24] Virulence [10] Biofilm formation [21] Cell metabolism [42]	ERY, NOV, TET, CHL, NAL, NOR, DXR, ACR, CV, ETBR, MB, R6G, TPP, BENZ, SDS, DOC, TRC [10, 152]
**MdsAB-MdsC/TolC**	Cell adhesion [24] Cell invasion [24, 44] Virulence [10, 44] Biofilm formation [21] Cell metabolism [45, 46]	CV, MB, ACR, R6G, NOV, ETBR, TPP, BENZ, SDS, Au [10, 44, 45]
**MdtABC-TolC**	Cell adhesion [24] Cell invasion [24] Virulence [10] Biofilm formation [21] Cell metabolism [39, 40]	NOV, SDS, DOC, Cu, Zn, sodium tungstate [10, 39, 40]
*** E. coli ***		
**AcrAB–TolC**	Cell proliferation [53, 57] Swarming motility [48] Biofilm formation [55] QSS [54] Cell metabolism [59, 68] Spontaneous mutation trigger [69]	CHL, TET, MIN, ERY, FQ, NAL, NOR, ENO, DXR, CIP, NOV, RIF, TMP, ACR, CV, ETBR, R6G, TPP, BENZ, SDS, DOC, bile salts, fatty acids, steroid hormones [47, 53, 58, 59, 155]
**Acr(A)D-TolC**	Biofilm formation [72] Cell metabolism [59, 75, 76]	Aminoglycosides, a very hydrophilic class of drugs, NOV, SDS, DOC, ATM, CAR, SB, Cu [47, 76, 156]
**AcrEF-TolC**	Cell proliferation [77] Biofilm formation [72] QSS [78] Cell metabolism [79]	DOC, ACR, ETBR, R6G, SDS, DOC [47]
**MdtEF-TolC**	Cell proliferation [80] Biofilm formation [72] Cell metabolism [75, 79, 82, 84]	TPP, ERY, NOV, DOX, CV, ETBR, SDS, DOC, BENZ, R6G, OXA, CLO [47, 84, 85]
**MdtABC-TolC**	Biofilm formation [55] Cell metabolism [75, 76, 87, 88]	NOV, DOC, Zn, flavonoids, sodium tungstate [47, 87, 88]
**CusABC**	Cell metabolism [90, 91]	Cu, Zn, Ag [89, 91]
*** P. aeruginosa ***		
**MexAB-OprM**	Cell invasion [106, 107] Biofilm formation [119] QSS [96, 99, 100, 102, 104] Virulence [99, 100, 106] Fitness cost adjustment [105]	Quinolones, macrolides, TETs, lincomycin, CHL, NOV, sulphonamides, TMP, β-lactams except imipenem [155, 157]
**MexCD-OprJ**	Swarming motility [112] Biofilm formation [115, 119] QSS [99] Virulence [111–114] Fitness cost adjustment [105, 112, 115, 117] Cell metabolism [116, 118]	Quinolones, macrolides, TETs, lincomycin, CHL, NOV, penicillins except CAR and SB, cephems except CAZ, flomoxef, meropenem, S-4661, biocides, dyes, detergents, organic solvents [155, 158]
**MexEF-OprN**	Swarming motility [121] Biofilm formation [121] QSS [97, 120, 121] Virulence [97, 111, 120, 121, 123] Fitness cost adjustment [105, 122] Cell metabolism [127–131]	CIP, LVX, EtBR, ACR, CHL, TET, TRI, TMP [159]
**MexXY-OprM**	Cell invasion [106] Fitness cost adjustment [105] Cell metabolism [132, 133, 139]	Quinolones, macrolides, TETs, lincomycin, CHL, aminoglycosides, penicillins except CAR, SB, cephems except cefsulodin and ceftazidime, meropenem, S-4661 [155]
**MexGHI-OpmD**	Cell proliferation [98, 140, 142, 143] Swarming motility [98, 140] Biofilm formation [142, 143] QSS [98, 140] Virulence [98, 140] Cell metabolism [118]	NOR, ETBR, ACR, R6G [160]
**CzcABC**	Cell metabolism [144, 145]	Cu, Zn [144, 145]
**MexPQ-OpmE**	–	Macrolides and FQ [161]
**MexVW-OprM**	–	FQ, TET, CHL, ERY, ETBR, ACR [162]
**MexJK-OprM/OpmH**	–	TRC, ERY [163]
**MexMN-OprM**	–	CHL, THI [161]
**TriABC-OpmH**	–	TRC [164]
**MuxABC-OpmB**	–	ATM, NOV, TET, ERY, kitasamycin, rokitamycin [156]

ACR, acriflavine; Ag, silverAMP, ampicillin; AMX, amoxicillin; ATM, aztreonam; Au, gold; BENZ, benzalkonium chloride; CAR, carbenicillin; CAZ, ceftazidime; CHL, chloramphenicol; CIP, ciprofloxacin; CLO, cloxacillin; CRO, ceftriaxone; CTX, cefoxitin; Cu, copper; CV, crystal violet; DOC, deoxycholic acid; DXR, doxorubicin; ENO, enoxacin; ERY, erythromycin; ETBR, ethidium bromide; FAM, cefamandole; FQ, fluoroquinolone; FUS, fusidic acid; LVX, levofloxacin; LZD, linezolid; MB, methylene blue; MIN, minocycline; NAF, nafcillin; NAL, nalidixic acid; NOR, norfloxacin; NOV, novobiocin; OXA, oxacillin; QSS, quorum sensing signal; R6G, rhodamine 6G; RIF, rifampicin; SB, sulbenicillin; SDS, sodium dodecyl sulfate; TET, tetracycline; TMP, trimethoprim; TPP, tetraphenylphosphonium bromide; TRC, triclosan; Zn, zinc.

### AcrAB–TolC

#### Introductory explanation

**Fig. 3.**
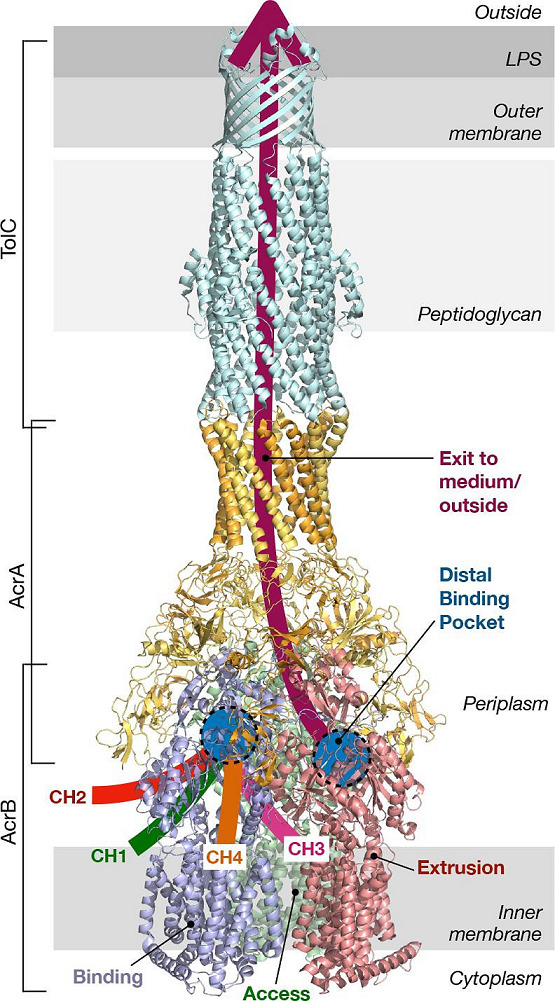
CryoEM structure of AcrAB–TolC. AcrAB–TolC is the best-studied RND-PAP-OMP tripartite system. AcrB is depicted as an asymmetrical homotrimer in the inner membrane. In the binding monomer (blue), the four main entrance channels are depicted as CH1, CH2, CH3 and CH4. In the extrusion monomer (red), the exit funnel is depicted in dark red, starting from the distal binding pocket (blue circle), continuing through the PAP-OMP tunnel structure, facilitating extruded molecule to be traversed out of the cells. PDB accession number used: 5O66 [165].

Acr(acriflavine, acridine)AB-Tol(tolerance)C is the principal constitutively expressed efflux system in *S. enterica* serovar Typhimurium [10]. This efflux system consists of three components: AcrA, AcrB and TolC (Fig. 3). Many homologues of this pump have been identified in other species [11]. AcrA is a periplasmic lipoprotein classified as a membrane fusion protein (MFP, also referred to as periplasmic adaptor protein or PAP) that bridges the outer and inner membrane proteins. AcrA is anchored to the inner membrane via a lipid motif [12]. AcrB is an inner cytoplasmic membrane RND-type efflux pump with 12 membrane-spanning α-helices [13]. TolC, an outer membrane tunnel, belongs to a family of envelope proteins found in all Gram-negative bacteria. TolC is essential for expelling many different compounds [14]. To investigate the physiological role of this pump, many experiments have been performed using genetic modifications that alter gene expression.

#### Cell adhesion and invasion

Experiments have been performed where genes encoding components of tripartite efflux pumps, including *acrA*, *acrB* and *tolC,* were directly manipulated (knockout or overexpression). Buckley *et al.* showed that mutants of the *S. enterica* strain SL1344 with deletions of *tolC* poorly adhered to and poorly invaded human intestinal epithelial cells (hECs) and mouse monocyte-derived macrophages. *acrB* gene deletion resulted in normal adhesion to hECs, but restricted invasion in hECs and macrophages. *In vivo*, the infection rates in birds of *acrB* and *tolC* mutant SL1344 strains were lower, owing to poor colonization and persistence in the avian gastrointestinal tract [15]. AcrA is also required for cell invasion in human intestinal cells, although its contribution might be less than that of AcrB and TolC [16]. Webber *et al.* conducted transcriptional analysis and showed that the absence of *acrB* and *tolC* caused widespread repression of chemotaxis and motility genes in these mutants, especially with the decreased motility of *acrB* mutants [17].

#### Virulence

Pathogenicity to host cells is influenced by virulence-related gene expression changes derived from the mutation of genes in the AcrAB–TolC system. During the inactivation of *acrB* or *tolC*, Webber *et al.* [17] also observed decreased gene expression at the transcriptional and protein levels of *S. enterica* pathogenicity island (SPI) 1 genes, which are involved in various components of the type III secretion system (T3SS) [18]. Conversely, Virlogeux-Payant *et al.*demonstrated a lower effect on invasion into hECs and virulence-related factors in an *acrB* knockout while observing disruptions in the expression of the SPI-1 genes *sipA*, *invF* and *hilA* in the absence of *tolC* [19]. These results are consistent with *in vivo* results by the same authors, where these *tolC* mutants infected chicks, notably in the intestine, and their parents [20]. The authors argued that an explanation for these differences with the outcome of Buckley *et al*. [15] in adhesion/invasion ability stems from differences between MDR and sensitive strains of *S. enterica* serovar Typhimurium.

#### Biofilm formation

Biofilm formation causing multidrug resistance has frequently been observed in *S. enterica *, as well as other bacterial species. Baugh *et al.* described, using 10 efflux pump (*acrB*, *tolC*, *acrD*, *acrEF*, *mdtABC*, *mdsABC*, *emrAB*, *mdfA*, *mdtK* and *macAB*) mutant strains in crystal violet (CV) biofilm assays, that an inability to form a competent biofilm resulted from the inactivation of individual multidrug resistance efflux systems of *S. enterica* [21]. Moreover, mutants of *S. enterica* serovar Typhimurium that lack *tolC* or *acrB*, but not *acrA*, had a compromised ability to form biofilms. One factor causing biofilm failure is the transcriptional repression of curli biosynthesis genes. Curli is a major component of the *S. enterica* biofilm extracellular matrix [22]. Conversely, Schlisselberg *et al*. reported that deletion of *acrB* alone or with *acrA* did not affect the ability of *S. enterica* serovar Typhimurium to form biofilms on polystyrene in Lysogeny Broth (LB) [23]. The main reason for this discrepancy is unknown, but the different experimental conditions under stressful environments might lead to contradictory findings.

#### Compensatory regulation mechanism

Without the *acrAB* pump, compensatory regulatory mechanisms are activated via other multidrug resistance pumps. The single deletion of *acrA* or *tolC* increased the expression of *acrB* because of the functional redundancy between RND efflux pumps. The deletion of *acrAB* increased the expression of seven other functional efflux pump genes (*acrF*, *acrD*, *mdsB*, *mdtB*, *macA*, *emrA* and *mdfA*) and vice versa in a compensatory signalling system. This compensation could be mediated through the coordinated upregulation of *ramA* and *marA*, global regulators of *S. enterica* pumps [24, 25]. Zhang *et al.* also indicated that the inactivation of each functional efflux pump gene reduced the adhesion and invasion abilities of *S. enterica* serovar Typhimurium into INT407 cells [24]. In contrast to strains lacking the *acrB* gene, Wang-Kan *et al.* investigated non-functional mutants of AcrAB–TolC pumps by mutating the single amino acid D408 [26]. The SL1344 *acrB* D408A mutant had the same native protein expression level of AcrB as the parental strain. However, the D408A mutant reduced the invasion into intestinal epithelial cells and macrophages *in vitro* and showed a lower survival rate *in vivo* in mouse and *Galleria mellonella* models. RNA-seq revealed the downregulation of SPI genes and the upregulation of stress response and flagellar motility genes. Unlike the loss of the AcrB protein, the loss of efflux function did not induce overexpression of other RND efflux pumps. Mutants containing pumps that do not function but are still present should be investigated, since this is likely to depict a more clinically realistic phenotype than gene deletion models.

#### RamA regulatory signalling

RamA, which binds directly to the promoter region of *acrAB*, is the most important regulator of *acrAB* expression in *S. enterica *. Bailey *et al.* indicated that highly overexpressed *ramA* led to increased expression of *acrAB, acrEF* and *tolC*. Decreased expression of multiple SPI-1 genes reduced adhesion to and survival within RAW 264.7 mouse macrophages, and decreased colonization of *Caenorhabditis elegans* was also seen. However, the inactivation of *ramA* led to increased expression of SPI-1 genes, reduced the expression of SPI-2 genes, and altered the expression of ribosomal biosynthesis genes and several amino acid biosynthesis pathways. Furthermore, disruption of *ramA* led to decreased survival within macrophages and attenuation in a BALB/c ByJ mouse model [27]. In another report, *ramA* expression promoted the development of ciprofloxacin (CIP) resistant mutants of *S. enterica* serovar Typhimurium (CVCC541 strain) with the increased expression level of *acrAB*. In contrast, the inhibition of *ramA* decreased the appearance of CIP-resistant mutants [28].

#### RamR regulatory signalling

*RamR*, located upstream of *ramA*, is a repressor of *ramA*. Disruption of *ramR* led to the increased expression of *ramA, acrAB* and *tolC* [27]. Giraud *et al.* investigated the expression levels of *acrAB-tolC* efflux-related genes and invasion-related genes in fluoroquinolone (FQ) resistant strains bearing natural mutations in the *ramRA* locus or FQ-susceptible strains, inducing overexpressed or deleted *ramR*. Unexpectedly, decreased expression of *acrAB-tolC* efflux-related genes and increased expression of invasion-related genes were observed in an FQ-resistant strain with *ramR* deletion, whereas the opposite expression patterns were observed in an FQ-susceptible strain with *ramR* deletion. The genetic background of the strains may influence these results. In contrast, an FQ-resistant strain (DT204 strain 102SA00) complemented with a wild-type *ramR* gene showed decreased *acrAB-tolC* expression and enhanced invasion ability as well as increases in *hilA, invA* and *sipA* transcript levels [29]. Structured binding to RamR by substrates such as berberine, CV, dequalinium, ethidium bromide (ETBR) and rhodamin 6G, and bile acids such as cholic and chenodeoxycholic acids was identified [30, 31].

#### Cell metabolism

In addition to the role of the AcrAB–TolC pump in drug transport and pathogen virulence, it also plays a role in cell metabolism through the export of host-derived substrates such as bile salts, fatty acids, and steroid hormones to survive within the stressful environment of the host. Bile-mediated activation of *acrB* and *tolC* occurred because of the direct binding of bile-activated *ramA* or bile binding to *ramR*, inducing the transcriptional derepression of *ramA* [32, 33]. Indole activates *ramA* transcription by repressing *ramR*, and overproduction of *ramA* causes increased *acrAB* expression [33, 34]. Additionally, indole represses SPI-1 genes, flagella production and the invasive activity of *S. enterica *, although this effect is independent of the *ramA* regulatory signal [35]. Recently, aside from the regulatory signalling of *ramRA*, carbon storage regulator A (CsrA) has been identified as a regulator of *acrAB* in the presence of indole [36]. Paraquat, a superoxide generator, induces *acrAB* expression dependent on *soxS* but not *ramA* [34].

### Acr(A)D-TolC

To function as an efflux pump, AcrA and TolC are necessary for AcrD and AcrB, although *acrA* is not located in the same operon as *acrD* (Table S2) [37]. The AcrD efflux pump is not simply a redundant system with AcrB but also has distinct physiological functions. Inactivation of *acrD* leads to changes in the expression of genes involved in basic metabolism, virulence and stress responses. The metabolite fumarate is required for switching of the direction of flagellum rotation. The perturbation of fumarate production by *acrD* deficiency is related to decreased swarming motility [38]. When *acrB* is deleted, *acrD* expression increases. We demonstrated that the two-component regulatory system *baeSR* induced the expression of *acrD* and *mdtABC* in response to indole, copper (Cu) and zinc (Zn) in strains lacking *acrB*. As a result, AcrD contributes to metal resistance in cooperation with MdtABC through the *baeSR* activation pathway [39]. In another report, there is a functional overlap between MdtA, AcrD and AcrB for resistance to sodium tungstate. The induction of *acrD* expression cloned in a plasmid into *baeR* mutation strains rescued cell survival in an environment containing tungstate [40].

### AcrEF-TolC

The AcrEF efflux pump of *S. enterica* serovar Typhimurium shows high sequence similarity to the AcrAB pump [41]. We reported that the histone-like nucleoid structuring protein (H-NS) modulated multidrug resistance through the repression of genes encoding the AcrEF multidrug efflux pump in *S.enterica* serovar Typhimurium [42]. There is not much information known regarding this pump.

### MdsAB-MdsC/TolC

*S. enterica *-specific Mds(multidrug transporter of *Salmonella *)AB functions with either MdsC or TolC for drug resistance, whereas other RND transporter systems only require TolC in *S. enterica* [43]. *S. enterica* infection of macrophages induces the upregulation of *mdsABC* expression and increases the intracellular bacterial number and host cell death. Strains with *mdsABC* deletion had decreased survival in infected macrophages. Additionally, overexpression of *mdsABC* leads to increased secretion of 1-palmitoyl-2-stearoyl-phosphatidylserine, affecting the ability of bacteria to invade and survive in host cells [44]. This pump system confers central resistance to gold (Au) stress and some antimicrobial dyes and oxidative stress-inducing agents [45]. MdsABC can also mediate the drug resistance induced by Au in a GolS-dependent manner in *acrAB*-deleted strains. In recent work by the same authors, they demonstrated that CpxR/CpxA, a cell envelope stress response system, enhanced the GolS-dependent transcription of *mdsABC* in the Au resistance mechanism at a neutral pH of 7.0 [46]. Phylogenetically, MdsB seems to be related to *P. aeruginosa* MexQ (Fig. 2).

### MdtABC-TolC

Like AcrD, Mdt(multidrug transporter)ABC contributes to metal tolerance through the *baeSR* activation pathway and to the waste disposal of tungstate from the cell [39, 40]. There is not much information known regarding this pump.

## The role of RND multidrug efflux pumps in *E. coli*

**Fig. 4.**
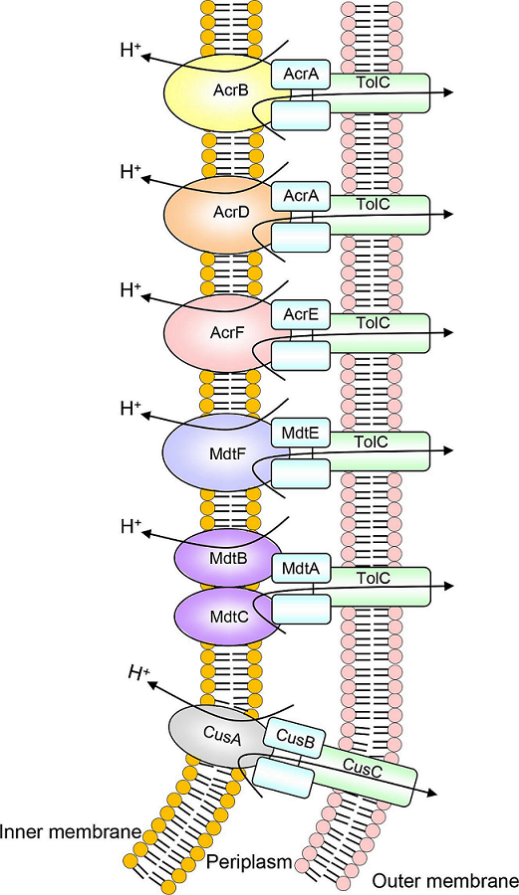
RND multidrug efflux pumps in *E. coli *.

*E. coli* is a major bacterial species in the environment and has many strains. Most *E. coli* are harmless, but some are pathogenic and can cause serious food poisoning to their hosts. Comprehensive expression cloning studies revealed that 20 functional drug efflux pumps are encoded on the *E. coli* K-12 chromosome, but most are not expressed under normal conditions [47]. These pumps in *E. coli* belong to five families: the MFS, RND, SMR, MATE and ABC (super) families. The following sections provide an overview of the six RND efflux pumps in *E. coli* (Fig. 4, Table 1). Many of the RND-type efflux pumps (AcrB, AcrF, AcrD and MdtBC) are phylogenetically closely related to those of *Salmonella* (Fig. 2) [7]. The outer membrane tunnel TolC is expressed from a separate operon *tolC-ygiABC* (Table S2).

### AcrAB–TolC

AcrAB–TolC is the main multidrug efflux pump in *E. coli* (Fig. 3). A strain lacking *acrB* had increased bacterial motility in LB medium supplemented with 0.3 % agar, consistent with the upregulation of motility and flagellar biosynthesis genes [48]. This efflux pump (AcrB) is the most studied RND-type efflux pump, of which the first crystal structure was solved in 2002 [49]. In-depth specific reviews can be found in [50–52].

#### Cell growth and QSS

Regarding cell growth, cells lacking *acrAB* had a delayed entry into the stationary phase with a higher cell density resulting from higher proliferation rates than wild-type strains. Conversely, overproduction of the pump led to a quick entry into the stationary phase with a lower cell density [53]. This might be affected by a QSS exported by AcrAB extracellularly, such as SdiA, which is homologous to the LuxR family of quorum-sensing transcription factors and upregulates *acrAB* [54]. That study showed that conditioned medium (CM) from cells overexpressing *acrAB* repressed the cell growth of cultured wild-type cells more than CM from wild-type cells [53]. Additionally, the expression level of RpoS, which is the stationary phase σ factor that controls the expression of multiple genes involved in adaptation and survival in the stationary phase, was upregulated when *acrAB* was overexpressed and decreased in *acrAB*-lacking strains. This suggests that the QSS is expelled at a higher level when *acrAB* is overexpressed and might upregulate RpoS, leading to cells entering the stationary phase with a lower cell density.

#### Biofilm formation

We confirmed that a single-deletion mutant of *acrB* normally produced biofilms in the *E. coli* wild-type strain TG1, in which the ability to form biofilms is particularly strong. However, our research showed that when *acrB* and *mdtABC* were deleted simultaneously, the cells did not demonstrate significant biofilm formation, despite growth being normal. Intriguingly, double-knockout mutants of *acrB* and *mdtABC* showed time-dependent biogenesis effects on biofilm maintenance; they maintained atypical biofilm at 4 h, but the biofilm gradually decreased and ultimately disappeared after 24 h. Thus, signalling factors such as the QSS by AcrB and MdtABC might be necessary to maintain the biofilm [55]. Bay *et al.* showed other findings that single deletions of *acrB*, *acrE* and *tolC* reduced biofilm growth and antimicrobial resistance, while deletions of other genes (*acrD*, *acrE*, *emrA*, *emrB*, *mdtK*, *emrE* and *mdtJ*) did not in *E. coli* K-12 strain [56].

#### Contact-dependent growth inhibition

In *E. coli *, AcrB is also involved in contact-dependent growth inhibition (CDI), which enables binding to neighbouring bacterial cells by direct cell-to-cell contact and delivery of protein toxins that inhibit cell growth. In the CDI mechanism, BamA is an outer membrane receptor, and AcrB functions downstream of BamA [57].

#### Regulatory signalling

The AcrAB–TolC pump can efflux many different classes of antibiotics, including chloramphenicol (CHL), tetracycline (TET), minocycline (MIN), erythromycin (ERY), FQ, nalidixic acid (NAL), norfloxacin (NOR), enoxacin (ENO), doxorubicin (DXR), CIP, novobiocin (NOV), rifampicin (RIF), trimethoprim (TMP), acriflavine (ACR), CV, ETBR, rhodamine 6G (R6G), tetraphenylphosphonium bromide (TPP), benzalkonium chloride (BENZ), sodium dodecyl sulfate (SDS) and deoxycholic acid (DOC) [47, 53, 58]. AcrAB–TolC works as an efflux pump for endogenous substrates such as bile salts, fatty acids and steroid hormones [7, 59]. The wide substrate specificity has been attributed to four channels: CH1 (low-molecular-mass drugs, β-lactams, NOV, phenicols), CH2 (high-molecular-mass drugs, macrolides, TETs), CH3 (planar aromatic cations) and CH4 (carboxylated drugs, including fusidic acid and β-lactams) (Fig. 3) [60–62] and the sub-sets of binding amino acids in two binding pockets of AcrB (the proximal binding pocket and the distal binding pocket, separated by a switch-loop) [63–65]. The three proteins of AcrAB–TolC are encoded in two separate operons, *acrAB* and *tolC-ygiAB* (Table S2). Major transcriptional regulators of AcrAB–TolC include MarA, SoxS and Rob as global regulators and AcrR as a local regulator [66, 67]. The activation of AcrAB pump by MarA and SoxS works via altering their own expression levels, whereas the upregulation of *acrAB* by Rob occurs via conformational alterations. The induction of the *acrAB* operon by bile salts and fatty acids is correlated with the binding of these effectors to pre-existing Rob [68]. As a high-level drug resistance mechanism, the latest literature shows that an increased level of AcrAB–TolC in *E. coli* results in spontaneous mutations following advanced DNA mismatch through lowered expression of the repair gene *mutS* [69].

#### Physiological function by the *tolC* gene

TolC comprises outer membrane components working with AcrAB, AcrD, AcrEF, MdtEF and MdtABC of RND multidrug efflux pumps in *E. coli *. TolC export enterobactins, ferric iron chelating compounds, through a two-step process with EntS. Mutant *E. coli* strains with the deletion of *tolC* or *entS* were growth-deficient and demonstrated insufficient enterobactin export in iron-depleted medium [70]. Evidence linking *tolC* expression to acidic pH resistance has also been reported. Deininger *et al.* indicated that for the *E. coli* K-12 W3110 strain, TolC was required for normal exponential growth, as well as acid survival (pH 2–7) in the stationary phase under aerobic conditions [71].

### Acr(A)D-TolC

Matsumura *et al.* showed that the deletion of *acrD* in *E. coli* K-12 BW25113 drastically decreased the cells’ biofilm formation ability, as did the deletion of *emrD*, *emrE*, *emrK*, *acrE* and *mdtE* [72]. In *E. coli *, the substrate specificity of AcrD is different from that of AcrB [47]. AcrD is also able to expel monobactams and anionic β-lactams, which AcrB could only weakly, or not at all [73]. Further, AcrD cannot expel macrolide ERY, TET and quinolones, which AcrB can [51]. Additionally, cholic acid and progesterone, endogenous factors that are exported via some pumps, including AcrD, have been identified [59]. A recent study elucidated the cryo-EM structure of AcrD, showing aminoglycoside gentamycin to be bound in the central cavity of the trimer, and it is postulated that aminoglycosides move through the monomer [74], seemingly similar to the entrance and location of CH3 in AcrB [60]. Several studies indicated that *acrD* is upregulated as well as *mdtABC* under the two-component systems BaeSR and CpxAR induced by indole, resulting in SDS resistance [75], and under the outer membrane lipoprotein NlpE, activating the CpxAR pathway during envelope stress, resulting in resistance to drugs such as oxacillin (OXA), nafcillin (NAF), aztreonam (ATM), DOC, cloxacillin (CLO), cefamandole (FAM), NOV and kanamycin (KAN), as well as Cu resistance [76].

### AcrEF-TolC

AcrF shares a high degree of homology with AcrB (Fig. 2). Lau *et al.* suggested that *acrEF* is expressed under normal growth conditions and plays a vital role in the regular maintenance of cell division in coordination with AcrAB. They found that increased expression of *acrA* in cells lacking *acrEF* results in a severe cell division defect that results in the formation of highly filamentous cells with abnormal nucleoids. Similar defects were observed in *tolC*-lacking strains, suggesting that functional AcrEF and TolC produced in the natural physiological state may be required to maintain the typical morphology of *E. coli* under the increased production of AcrA [77]. In addition to the AcrAB efflux pump, *acrEF* expression is dramatically upregulated in cells overproducing the quorum signal receptor SdiA [78]. Our research showed that the inactivation of H-NS, which is involved in bacterial chromosome condensation, increased the expression of *acrEF* and *mdtEF* and the occurrence of drug resistance [79].

### MdtEF-TolC

The transcriptional expression of *mdtEF*, but not of other pumps, was enhanced by the stationary phase σ factor RpoS and the RpoS-dependent signalling pathway, Hfq, GadY and GadX, during the stationary phase. This pump’s upregulation maintained the normal cell growth of the *E. coli* MC4100 strain during CV treatment [80]. Additionally, indole in *E. coli* conferred R6G resistance through the induction of *mdtEF* gene expression mediated by GadX [75]. Furthermore, we found that the expression of *mdtEF* was significantly enhanced by the induction of phosphotransferase system sugars such as N-acetyl-d-glucosamine. In this signalling pathway, the cAMP–Crp complex, a catabolite repressor, decreased with the induction of *mdtEF* expression [81]. Zhang *et al.* described the importance of upregulated *mdtEF* under anaerobic conditions, an environmental signature of the mammalian gut, and the anaerobic microhabitat of bacteria in nature. This pump protects from nitrosative damage by expelling nitrosyl indole derivatives out of *E. coli* K-12, resulting in normal cell growth under anaerobic conditions [82]. The expression of *mdtEF* is induced by the two-component system regulator, EvgA, which is an acid resistance regulon [83]. Other research by Masuda *et al.* described that deletion of *mdtEF* completely abolished multidrug resistance in an *acrB*-deletion mutant caused by *evgA* overexpression, suggesting that MdtEF induced by EvgA functions as a multidrug transporter against ERY, NOV, DOX, CV, ETBR, SDS, DOC, BENZ and R6G [84]. They also showed that *evgA* overexpression conferred acid resistance to exponentially growing cells. Thus, a regulatory function of MdtEF for acid homeostasis could be inferred. Finally, we found that the small non-coding RNA DsrA significantly increased the expression of *mdtE* in a random shotgun cloning experiment. DsrA decreases susceptibility to OXA, CLO, ERY, R6G and NOV, mainly via RpoS pathway-mediated upregulation of *mdtEF* [85].

### MdtABC-TolC

We found that the MdtABC system comprises the transmembrane MdtB/MdtC heteromultimer and the MdtA membrane fusion protein. Additionally, the two-component signal transduction system BeaR enables the upregulation of *mdtABC* [86]. MdtABC is relevant to Zn homeostasis as part of the BaeSR regulatory system, maintaining a low cellular Zn concentration upon exposure to high extracellular Zn. An *mdtC*-deleted mutant in *E. coli* decreased the cell growth rate at a high cell density in the presence of a high Zn concentration. This mutant failed to perform Zn detoxification with a significant increase in the total intracellular Zn concentration after zinc shock [87]. Flavonoids and sodium tungstate have also been reported as natural substrates of the MdtABC efflux pump [88].

### CusABC

Cus(Cu sensor)ABC is an RND-type transporter belonging to the heavy metal efflux (HME) subfamily (Fig. 2) that plays a crucial role in Cu resistance, similar to the function of MdtABC in Zn resistance [89]. This pump transports Cu into the cell by a stepwise shuttle mechanism via a series of methionine pairs [90]. Additionally, this pump exports silver (Ag) [91]. Not much information is known regarding this pump.

## The role of RND multidrug efflux pumps in *P. aeruginosa*

**Fig. 5.**
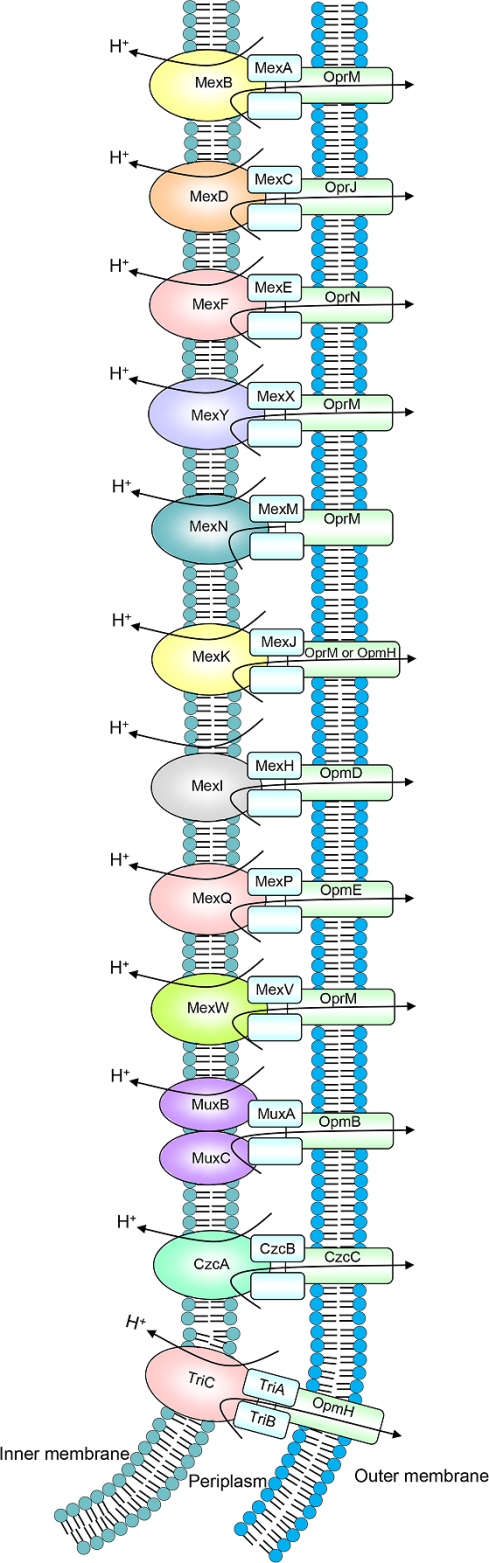
RND multidrug efflux pumps in *P. aeruginosa *.

*P. aeruginosa* is an important opportunistic human pathogen that is responsible for life-threatening infections that are difficult to eradicate in immunocompromised patients with AIDS, cystic fibrosis (CF), ventilation-associated pneumonia, chronic urinary tract infections due to catheters, or burn wounds. *P. aeruginosa* clinical and experimental strains show high frequencies of mutations (e.g. due to the high exposure to oxidative stress in the lungs of CF patients). The strong capacity of this pathogen severely compromises the treatment of these infections through the complex interplay of intrinsic and mutation-driven acquired resistance. Twelve systems in the RND superfamily capable of secreting antibiotics or antimicrobial products have been functionally described in *P. aeruginosa* [92–94]: MexAB-OprM, MexCD-OprJ, MexEF-OprN, MexGHI-OpmD, MexJK-OprM/OpmH, MexMN-OprM, MexPQ-OpmE, MexVW-OprM, MexXY-OprM, CzcABC, TriABC-OpmH and MuxABC-OpmB (Fig. 5, Table 1). Compared to *Salmonella* and *E. coli *, *P. aeruginosa *’s outer membrane components are typically expressed from the same operon as their corresponding RND-PAP genes, with the exceptions of MexXY, MexMN, MexJK, MexVW and TriABC, where OprM is expressed from the separate *mexABoprM* operon and OprH from the *oprHphoPQ* operon (Table S2).

### MexAB-OprM

The operon encoding Mex(multiple efflux)AB-Opr(outer membrane protein)M was found as genes with increased expression in siderophore-deficient mutants that can grow on iron-deficient minimal medium in 1993 by Poole *et al.* They found that this operon was involved in drug sensitivity as well as in the secretion of the siderophore pyoverdine [95]. Of the multi-efflux pumps in *P. aeruginosa *, only MexAB-OprM is constitutively expressed in wild-type cells and is a major contributor toward the high basal level of antibiotic resistance.

#### QSS and virulence

QSS is an important factor for cell-to-cell communications in bacteria. 3-oxo-C12-homoserine lactone (3OC12-HSL) can passively diffuse, but the rate is much slower than C4-homoserine lactone (C4-HSL), which is another freely diffusible AHL signal [96]. MexAB and potentially MexCD, MexEF and MexGHI assist the diffusion of the 3OC12-HSL signal from *P. aeruginosa* [96–99]. Because QSS communications between bacteria, including *P. aeruginosa *, control virulence factors and biofilm formation, secreting 3OC12-HSL is a critical function in the physiological role of the MexAB-OprM efflux pump. *P. aeruginosa* has two LuxI/LuxR pairs, LasI/LasR and RhlI/RhlR, which catalyze the last step in the synthesis of 3OC12-HSL and C4-HSL, respectively. *mexAB-oprM* overexpression induced by a *nalB* mutation compromised the production of 3OC12-HSL, decreased the expression of LasI/LasR-dependent virulence factors, such as pyocyanin, casein protease and elastase, and attenuated virulence in a *C. elegans* model [99, 100]. Conversely, clinical isolates of *P. aeruginosa* strains lacking a functional LasI/LasR system are less virulent in animal infection models, indicating their function as a virulence activator via QSS systems [101]. In *mexAB*-knockout mutant strains, 3OC12-HSL was not secreted, and LasR was upregulated by the accumulation of 3OC12-HSL inside cells [102]. While the exogenous addition of 3OC12-HSL had little or no effect on *mexAB-oprM* expression, C4-HSL enhanced *mexAB-oprM* expression in the stationary phase. The upregulation mechanism was not mediated by MexR, a negative regulator of MexAB-OprM [103]. MexAB-OprM can not only transport 3OC12-HSL but a small set of naturally occurring 3OC-HSLs (with 8–14 carbon acyl tails) and respond to QSS cross-talk from other species of bacteria [104].

#### Metabolic rewiring

To acquire antibiotic resistance, bacteria must pay a fitness cost. Recently, efflux pumps, including not only MexAB-OprM but MexCD-OprJ, MexEF-OprN and MexXY, have been recognized as contributing to the fitness cost management of environmental adaptation using a metabolic rewiring system [105]. Pacheco *et al.* found that overexpression of these four efflux pumps led to the rewiring of bacterial metabolism to generate an energy that is utilized for avoiding the fitness cost of by the acquisition of resistance due to overexpression of RND efflux pumps.

#### Virulence effect *in vivo*

Hirakata *et al.* reported that fluctuations in the MexAB expression levels contribute to virulence in a *mexAB-oprM*-deletion mutant or via inhibition of MexAB in the *P. aeruginosa* PAO1 strain, resulting in a significantly decreased capacity to invade or transmigrate across Madin–Darby canine kidney (MDCK) cells [106]. Furthermore, a *mexAB-oprM*-deletion strain could not kill a septicaemia murine model, in contrast to wild-type cells. Since the invasiveness of the *mexAB-oprM*-deletion mutant was restored by adding culture supernatant from MDCK cells infected with the wild-type, the authors suggested that *P. aeruginosa* exports invasion determinants using theMexAB-OprM system [107]. However, the essential involvement of QSSs such as C4-HSL and 3OC12-HSL was not apparent in additional experiments, so the specific molecule involved in virulence remains unidentified [108].

#### Biofilm formation

*P. aeruginosa* biofilm is intrinsically resistant to antimicrobial chemotherapies, and their formation is required for chronic colonization in human tissue. In the early 2000s, reports showed that overexpression or knockout of *mexAB-oprM*, *mexCD-oprJ*, *mexEF-oprN* and *mexXY* were uncorrelated with the level of antibiotic resistance in biofilms [109]. The interaction between efflux pumps and biofilm formation has been re-evaluated in the past decade. Liao *et al*. indicated that biofilm of double-mutant strains of *mexAB-oprM* and *mexEF-oprM* were more susceptible to five antibiotics, KAN, NOR, TET, TMP and tobramycin (TOB). Conversely, the overexpression of either efflux pump partly restored resistance in strains lacking *brlR*, a biofilm-specific MerR-type transcriptional regulator [110].

### MexCD-OprJ

MexCD-OprJ comprises RND efflux pump MexD, the outer membrane protein OprJ and a periplasmic adaptor protein MexC that bridges the other two components.

#### Virulence

The relationship between *mexCD-oprJ* expression in *P. aeruginosa* and its virulence has been examined in several studies. Linares *et al.* indicated that overexpression of either *mexCD-oprJ* or *mexEF-oprN* resulted in the impairment of a T3SS in *P. aeruginosa* due to the transcriptional reduction of T3SS genes such as *pcrv*, *exoT* and *exoS* [111]. *P. aeruginosa* uses T3SS to carry virulent compounds directly into the cytoplasm of the mammalian host cell.

#### Physiological effect of *nfxB* mutant

Studies have investigated the effects of *mexCD* overexpression by utilizing a strain with loss-of function mutations in *nfxB*, encoding a negative regulator of the *mexCD-oprJ* genes. The *nfxB* mutant was rapidly out-competed in growth in a mixed culture with wild-type cells during the stationary phase and showed motility impairment (swimming, swarming and twitching), and the reduced production of virulence factors (siderophores, rhamnolipid, secreted protease and pyocyanin) [112]. Intriguingly, Martinez-Ramos *et al.* demonstrated that C3 opsonization was increased, and infection of mouse lung tissues was attenuated in the *nfxB* mutant strain of *P. aeruginosa *, supporting the notion that MexCD-OprJis responsible for the interaction with the mammalian host immune system [113]. *nfxB* mutants are rarely encountered in clinical samples due to poorly designed screening methodologies. Jeannot *et al*. only found 4 *nfxB* mutants (3.6%) in 110 nonreplicated clinical isolates after CIP treatment. These mutants had *mexCD-oprJ* upregulation and impaired colony size with strain-specific variation in virulence factors [114]. The emergence of *nfxB* mutants after CIP treatment was recently investigated in real time with an a*nfxB*-GFP tracing experiment under conditions enabling biofilm formation. The results showed that *nfxB* mutants had an increased biofilm growth ability compared to wild-type cells, probably via the upregulation of *mexCD-oprJ* in biofilms. Moreover, *nfxB* mutants emerged *de novo* in the biofilm during CIP treatment from filamentous cells due to the stress response induced by CIP [115].

#### Physiological effect of *nfxB* mutant with *mutT*/*mutY*/*mutM* deletions

MexCD-OprJ is expressed from the envelope stress-inducible multidrug efflux operon *mexCD-oprJ* of *P. aeruginosa *. Single-inactivation mutants of *mutT* and *mutY*, which are DNA repair genes that counter oxidative damage after exposure to polymorphonuclear leukocytes, significantly promoted the expression of *mexCD-oprJ* after CIP stimulation, leading to bacterial reactive oxygen species (ROS) production, suggesting a mechanism of acquiring antimicrobial resistance [116]. Furthermore, for *mutY* and *mutM* double-inactivation mutants, mutations in the regulator *nfxB,* led to hyperexpression of *mexCD-oprJ*, and these mutants showed out-competed growth compared to wild-type PAO1 strains. Thus, the hyperexpression of MexCD-OprJ was related to the mechanism of CIP resistance [117].

#### The effect of immune molecules

MexCD-OprJ and MexGHI-OpmD play defensive roles in protection against host defence immune peptides. Strempel *et al*. indicated that exogenous LL-37, a major human host defence peptide, promoted the expression of *mexCD-oprJ* and *mexGHI-opmD*, the production of virulence factors (such as toxic metabolites and proteases), and adaptive resistance against antibiotics such as CIP and gentamicin in *P. aeruginosa* strain PAO1 [118].

#### Azithromycin resistance

In addition to CIP treatment, macrolide azithromycin (AZM) is an antibiotic used for treating chronic *P. aeruginosa* infections in CF, but CIP resistance is frequently observed. If either *mexAB-oprM* or *mexCD-oprJ* is expressed, biofilm formation occurs in the presence of AZM. However, the deletion of both operons halts biofilm formation under the same conditions. This indicates that either of the pumps can confer resistance to AZM during biofilm development [119].

### MexEF-OprN

#### QSSs and virulence of the *nfxC* mutant

MexEF-OprN is overexpressed when its negative regulator *nfxC* is disrupted. *nfxC-*deletion strains overexpressing MexEF-OprN produced lower levels of extracellular C4-HSL and virulence factors such as pyocyanin, elastase and rhamnolipids [97]. In addition to QSSs such as C4-HSL and 3OC12-HSL, 3,4-dihydroxy-2-heptylquinoline, known as the *Pseudomonas* quinolone signal (PQS), is another QSS molecule. The production of PQS depends on the transcriptional regulators PqsR/PqsA-D/PqsH. 4-hydroxy-2-heptylquinoline (HHQ) is the immediate PQS precursor before final catalysis by PqsH. Olivares *et al.* demonstrated that the overexpression of the MexEF-OprN delayed the production of PQS. This was probably caused by the extrusion of kynurenine, a PQS and HHQ precursor, through the MexEF-OprN pump. Overexpression of MexEF-OprN also impaired virulence factors such as T3SS and type VI secretion system (T6SS) at the transcriptional level and enhanced the survival of the infected host, *C. elegans*, *in vivo* [120]. Additionally, the activation of MexEF-OprN in *P. aeruginosa* PA14 strains with mutations in the suppressor *mexS* also reduced swarming motility, virulence factor pyocyanin production and biofilm formation. In this condition, decreased PQS concentration and increased HHQ were observed in overexpressed *mexEF-oprN*, suggesting that MexEF-OprN exported HHQ before PQS was synthesized, and the efflux of QSS by MexEF-OprN may be a reason for the reduced virulence [121].

#### Physiological effect of variation in the regulatory signal

*mexEF-oprN* expression is enhanced by the functional activator protein MexT, a LysR family transcriptional regulator. Recently, Oshri *et al*. showed that *mexT*-null mutants inhibited MexEF-OprN activity and enhanced cell growth, although an independent function of MexT was also confirmed separately from the dependent effect of the MexEF-OprN pump [122], consistent with previous reports including the involvement of T3SS [123]. The MexS protein also influences MexEF-OprN efflux activity in interaction with MexT [124]. Upstream signals of MexS/MexT, ParS and ParR were proposed, and both the *parS* and *parR* mutants induced a decrease in the MexEF pump and upregulated the virulence factor pyocyanin and enhanced swarming motility [125]. Another negative regulatory factor, AmpR, a member of the LysR family, also regulates non-β-lactam antibiotic resistance by activating the expression of theMexEF-OprN efflux pump. Although the contribution of MexEF-OprN was not determined, *ampR*-deletion mutants showed enhanced biofilm formation, decreased QSS-regulated acute virulence factors and attenuated virulence in a *C. elegans* model in parallel with upregulated transcription of *mexEF-oprN* genes [126].

#### Stress response

MexEF-OprN plays a central role in pathogen response to several stresses, such as electrophilic and metabolic compounds, disulfide, and oxidative and nitrosative stress, affecting intracellular energy homeostasis. Under toxic electrophilic molecule stress (by glyoxal, methylglyoxal and cinnamaldehyde) in PA14 strains, the CmrA pathway, a regulator of the AraC family, is strongly activated. MexEF-OprN is upregulated through MexS and MexT activation, suggesting that a protection system counteracts these stressful agents [127]. MexEF-OprN also offered protection against endogenous metabolic stressors by incubation on a nutrient-rich medium without antibiotics. This protection occurs in a MexT-independent manner, although the mechanism remains unresolved [128]. Disulfide stress induced by diamide reaction with free thiols is a subcategory of oxidative stress causing thiol-disulfide imbalances, leading to stress-inducing redox imbalances because of naturally occurring electrophiles. Fargier *et al*. reported that MexEF-OprN is induced by MexT in the presence of diamide, which increased susceptibility to disulfide stress in redox control [129]. Under hypoxic conditions, only the MexEF-OprN efflux pump system, but neither MexAB-OprM nor MexCD-OprJ, is upregulated in *P. aeruginosa* clinical strains and ATCC27853. Since this condition made these cells resistant to antibiotic drugs, MexEF-OprN was suggested as a strong candidate for mediating this phenotype [130]. MexEF-OprN is likely to be induced by nitrosative stress. *mexEF-oprN* overexpression has also been observed to be MexT-dependent, by using *s*-nitrosoglutathione as a source of nitrosative stress without antibiotics [131].

### MexXY-OprM

The MexXY-OprM efflux pump complex functions in response to the impairment of ribosome function or protein synthesis, or antibiotics that perturb ribosome function. Either methionyl-tRNA^fmet^formyltransferase (*fmt*) mutation or *folD* mutation upregulated expression of *mexXY* after impaired cell growth due to the impairment of protein synthesis [132]. Various ribosome inhibitors (e.g. CHL, TET, macrolides and aminoglycosides) induced the expression of *mexXY-oprM* in a concentration-dependent manner in the *P. aeruginosa* PAO1 strain. MexZ-independent MexXY activation in the presence of TET, CHL and spectinomycin was recognized as MexXY was upregulated in a PAO1 mutant with *mexZ* deficiency [133]. Aside from MexZ and its upstream ArmZ regulatory signal [134], ParRS, AmgRS and SuhB have been reported as candidate regulatory systems for MexXY-OprM during ribosome inhibition by antibiotics [135–138]. MexXY-OprM is also inducible by oxidative stress. Since ROS is present in CF lungs and *P. aeruginosa* infection due to chronic inflammation, the significant resistance to aminoglycosides is accelerated by the upregulation of MexXY through ArmZin the presence of high ROS levels [139].

### MexGHI-OpmD

MexGHI-OpmD confers vanadium resistance, QSS homeostasis and virulence production in *P. aeruginosa *. Adendekerk *et al*. demonstrated that either *mexI* or *opmD* deletion accumulated intracellular QSS factors such as C4-HSL, 3OC12-HSL and PQS. Both single-deletion mutants showed impaired cell growth, swarming motility and production of virulence factors such as elastase, rhamnolipids and pyocyanin. Notably, the attenuation of virulence was confirmed *in vivo* in rat and plant models infected with these mutants of *P. Aeruginosa* [98, 140]. Phenazines include phenazine-1-carboxylate (PCA), the intermediate 5-Me-PCA, and pyocyanin (5-N-methyl-1-hydroxyphenazine), which are in the phenazine biosynthetic pathway of redox-cycling antibiotics that affect redox homeostasis. Research by Dietrich *et al.* demonstrated that *mexGHI-opmD* expression was promoted by endogenous synthesized 5-Me-PCA and phenazines through the redox-active transcription factor SoxR in the *P. aeruginosa* PA14 strain. 5-Me-PCA is required for biofilm morphogenesis with the upregulation of MexGHI, resulting in a more reduced cellular phenazine pool [141–143]. Since MexG was not required for 5-Me-PCA efflux, the function of this protein is unknown [142].

### CzcABC

Zn and Cu are required for bacterial growth, but in excess they jeopardize normal survival due to toxicity. The regulation of intracellular heavy metals is achieved via the Czc(cadmium, zinc and cobalt)ABC efflux system. When *P. aeruginosa* cells were treated with Zn, the expression of *czcABC* was upregulated through the activation of the two-component system CzcRS for intrinsic Zn resistance [144]. In the presence of Cu, the transcription of *czcABC* was enhanced with CzcRS upregulation, leading to resistance to not only Cu but also Zn [145].

### Other efflux pumps

The physiological functions of MexJK-OprM/OpmH, MexMN-OprM, MexPQ-OpmE, MexVW-OprM, Tri(triclosan)ABC-OpmH and MuxABC-OpmB remain unknown. Not much information is known regarding these pumps.

## Conclusion and future perspectives

We reviewed the physiological and drug resistance roles of RND-type drug efflux pump systems in *S. enterica *, *E. coli* and *P. aeruginosa *. In this review, we categorized how efflux pumps work physiologically in virulence, biofilm formation and cell metabolism. Intriguingly, not only major efflux pumps, such as AcrAB and MexAB, but also other pumps are recognized for their important capacity to help bacteria survive harsh environments in mammalian host cells. However, several factors remain unknown, including the functional role of several types of efflux pumps and the detailed mechanism underlying their physiological roles. In future work, we aim to comprehensively investigate these physiological roles. Additionally, we described the substrates of each efflux pump complex, which is important for the multidrug resistance of Gram-negative pathogens. Since some multidrug efflux pumps contribute to virulence in host cells, drug development to prevent drug resistance and virulence should be further investigated. Although several research groups have attempted to develop efficient efflux pump inhibitors (EPIs), clinically useful inhibitors are not yet available. In the future, we hope EPI candidates inhibiting multidrug efflux pumps come into the market, helping to prevent and cure MDR infections.

## Supplementary Data

Supplementary material 1

## References

[1] Reygaert WC (2018). An overview of the antimicrobial resistance mechanisms of bacteria. AIMS Microbiol.

[2] Hassan KA, Liu Q, Henderson PJF, Paulsen IT (2015). Homologs of the *Acinetobacter baumannii* AceI transporter represent a new family of bacterial multidrug efflux systems. mBio.

[3] Kornelsen V, Kumar A (2021). Update on multidrug resistance efflux pumps in *Acinetobacter* spp. Antimicrob Agents Chemother.

[4] Nishino K, Nikaido E, Yamaguchi A (2009). Regulation and physiological function of multidrug efflux pumps in *Escherichia coli* and *Salmonella*. Biochim Biophys Acta.

[5] Putman M, van Veen HW, Konings WN (2000). Molecular properties of bacterial multidrug transporters. Microbiol Mol Biol Rev.

[6] Henderson PJF, Maher C, Elbourne LDH, Eijkelkamp BA, Paulsen IT (2021). Physiological functions of bacterial “Multidrug” efflux pumps. Chem Rev.

[7] Zwama M, Yamaguchi A, Nishino K (2019). Phylogenetic and functional characterisation of the *Haemophilus influenzae* multidrug efflux pump AcrB. Commun Biol.

[8] Górecki K, McEvoy MM (2020). Phylogenetic analysis reveals an ancient gene duplication as the origin of the MdtABC efflux pump. PLoS One.

[9] Yen MR, Chen JS, Marquez JL, Sun EI, Saier MH (2010). Multidrug resistance: Phylogenetic characterization of superfamilies of secondary carriers that include drug exporters. Methods Mol Biol.

[10] Nishino K, Latifi T, Groisman EA (2006). Virulence and drug resistance roles of multidrug efflux systems of *Salmonella enterica* serovar typhimurium. Mol Microbiol.

[11] Piddock LJV (2006). Clinically relevant chromosomally encoded multidrug resistance efflux pumps in bacteria. Clin Microbiol Rev.

[12] Ma D, Cook DN, Alberti M, Pon NG, Nikaido H (1993). Molecular cloning and characterization of acrA and acrE genes of *Escherichia coli*. J Bacteriol.

[13] Zgurskaya HI, Nikaido H (1999). Bypassing the periplasm: Reconstitution of the AcrAB multidrug efflux pump of *Escherichia coli*. Proc Natl Acad Sci U S A.

[14] Eswaran J, Hughes C, Koronakis V (2003). Locking TolC entrance helices to prevent protein translocation by the bacterial type I export apparatus. J Mol Biol.

[15] Buckley AM, Webber MA, Cooles S, Randall LP, La Ragione RM (2006). The AcrAB-TolC efflux system of *Salmonella enterica* serovar Typhimurium plays a role in pathogenesis. Cell Microbiol.

[16] Blair JMA, La Ragione RM, Woodward MJ, Piddock LJV (2009). Periplasmic adaptor protein AcrA has a distinct role in the antibiotic resistance and virulence of *Salmonella enterica* serovar Typhimurium. J Antimicrob Chemother.

[17] Webber MA, Bailey AM, Blair JMA, Morgan E, Stevens MP (2009). The global consequence of disruption of the AcrAB-TolC efflux pump in *Salmonella enterica* includes reduced expression of SPI-1 and other attributes required to infect the host. J Bacteriol.

[18] Collazo CM, Galán JE (1997). The invasion-associated type-III protein secretion system in *Salmonella*--a review. Gene.

[19] Virlogeux-Payant I, Baucheron S, Pelet J, Trotereau J, Bottreau E (2008). TolC, but not AcrB, is involved in the invasiveness of multidrug-resistant *Salmonella enterica* serovar Typhimurium by increasing type III secretion system-1 expression. Int J Med Microbiol.

[20] Baucheron S, Mouline C, Praud K, Chaslus-Dancla E, Cloeckaert A (2005). TolC but not AcrB is essential for multidrug-resistant *Salmonella enterica* serotype Typhimurium colonization of chicks. J Antimicrob Chemother.

[21] Baugh S, Ekanayaka AS, Piddock LJV, Webber MA (2012). Loss of or inhibition of all multidrug resistance efflux pumps of *Salmonella enterica* serovar Typhimurium results in impaired ability to form a biofilm. J Antimicrob Chemother.

[22] Baugh S, Phillips CR, Ekanayaka AS, Piddock LJV, Webber MA (2014). Inhibition of multidrug efflux as a strategy to prevent biofilm formation. J Antimicrob Chemother.

[23] Schlisselberg DB, Kler E, Kisluk G, Shachar D, Yaron S (2015). Biofilm formation ability of *Salmonella enterica* serovar Typhimurium acrAB mutants. Int J Antimicrob Agents.

[24] Zhang C-Z, Chen P-X, Yang L, Li W, Chang M-X (2018). Coordinated expression of acrAB-tolC and eight other functional efflux pumps through activating ramA and marA in *Salmonella enterica* serovar typhimurium. Microb Drug Resist.

[25] Blair JMA, Smith HE, Ricci V, Lawler AJ, Thompson LJ (2015). Expression of homologous RND efflux pump genes is dependent upon AcrB expression: Implications for efflux and virulence inhibitor design. J Antimicrob Chemother.

[26] Wang-Kan X, Blair JMA, Chirullo B, Betts J, La Ragione RM (2017). Lack of acrB efflux function confers loss of virulence on *Salmonella enterica* serovar typhimurium. mBio.

[27] Bailey AM, Ivens A, Kingsley R, Cottell JL, Wain J (2010). RamA, a member of the AraC/XylS family, influences both virulence and efflux in *Salmonella enterica* serovar Typhimurium. J Bacteriol.

[28] Sun Y, Dai M, Hao H, Wang Y, Huang L (2011). The role of ramA on the development of ciprofloxacin resistance in *Salmonella enterica* serovar typhimurium. PLoS One.

[29] Giraud E, Baucheron S, Virlogeux-Payant I, Nishino K, Cloeckaert A (2013). Effects of natural mutations in the ramRA locus on invasiveness of epidemic fluoroquinolone-resistant *Salmonella enterica* serovar Typhimurium isolates. J Infect Dis.

[30] Yamasaki S, Nikaido E, Nakashima R, Sakurai K, Fujiwara D (2013). The crystal structure of multidrug-resistance regulator RamR with multiple drugs. Nat Commun.

[31] Yamasaki S, Nakashima R, Sakurai K, Baucheron S, Giraud E (2019). Crystal structure of the multidrug resistance regulator RamR complexed with bile acids. Sci Rep.

[32] Baucheron S, Nishino K, Monchaux I, Canepa S, Maurel M-C (2014). Bile-mediated activation of the acrAB and tolC multidrug efflux genes occurs mainly through transcriptional derepression of ramA in *Salmonella enterica* serovar Typhimurium. J Antimicrob Chemother.

[33] Nikaido E, Yamaguchi A, Nishino K (2008). AcrAB multidrug efflux pump regulation in *Salmonella enterica* serovar Typhimurium by RamA in response to environmental signals. J Biol Chem.

[34] Nikaido E, Shirosaka I, Yamaguchi A, Nishino K (2011). Regulation of the AcrAB multidrug efflux pump in *Salmonella enterica* serovar Typhimurium in response to indole and paraquat. Microbiology.

[35] Nikaido E, Giraud E, Baucheron S, Yamasaki S, Wiedemann A (2012). Effects of indole on drug resistance and virulence of *Salmonella enterica* serovar Typhimurium revealed by genome-wide analyses. Gut Pathog.

[36] Ricci V, Attah V, Overton T, Grainger DC, Piddock LJV (2017). CsrA maximizes expression of the AcrAB multidrug resistance transporter. Nucleic Acids Res.

[37] Yamasaki S, Nagasawa S, Hayashi-Nishino M, Yamaguchi A, Nishino K (2011). AcrA dependency of the AcrD efflux pump in *Salmonella enterica* serovar Typhimurium. J Antibiot.

[38] Buckner MMC, Blair JMA, La Ragione RM, Newcombe J, Dwyer DJ (2016). Beyond Antimicrobial Resistance: Evidence for a distinct role of the AcrD efflux pump in *Salmonella* biology. mBio.

[39] Nishino K, Nikaido E, Yamaguchi A (2007). Regulation of multidrug efflux systems involved in multidrug and metal resistance of *Salmonella enterica* serovar Typhimurium. J Bacteriol.

[40] Appia-Ayme C, Patrick E, Sullivan MJ, Alston MJ, Field SJ (2011). Novel inducers of the envelope stress response BaeSR in *Salmonella* Typhimurium: BaeR is critically required for tungstate waste disposal. PLoS One.

[41] Eaves DJ, Ricci V, Piddock LJV (2004). Expression of acrB, acrF, acrD, marA, and soxS in *Salmonella enterica* serovar Typhimurium: Role in multiple antibiotic resistance. Antimicrob Agents Chemother.

[42] Nishino K, Hayashi-Nishino M, Yamaguchi A (2009). H-NS modulates multidrug resistance of *Salmonella enterica* serovar Typhimurium by repressing multidrug efflux genes acrEF. Antimicrob Agents Chemother.

[43] Horiyama T, Yamaguchi A, Nishino K (2010). TolC dependency of multidrug efflux systems in *Salmonella enterica* serovar Typhimurium. J Antimicrob Chemother.

[44] Song S, Lee B, Yeom J-H, Hwang S, Kang I (2015). MdsABC-mediated pathway for pathogenicity in *Salmonella enterica* serovar Typhimurium. Infect Immun.

[45] Pontel LB, Audero MEP, Espariz M, Checa SK, Soncini FC (2007). GolS controls the response to gold by the hierarchical induction of *Salmonella*-specific genes that include a CBA efflux-coding operon. Mol Microbiol.

[46] Cerminati S, Giri GF, Mendoza JI, Soncini FC, Checa SK (2017). The CpxR/CpxA system contributes to *Salmonella* gold-resistance by controlling the GolS-dependent gesABC transcription. Environ Microbiol.

[47] Nishino K, Yamaguchi A (2001). Analysis of a complete library of putative drug transporter genes in *Escherichia coli*. J Bacteriol.

[48] Ruiz C, Levy SB (2014). Regulation of acrAB expression by cellular metabolites in *Escherichia coli*. J Antimicrob Chemother.

[49] Murakami S, Nakashima R, Yamashita E, Yamaguchi A (2002). Crystal structure of bacterial multidrug efflux transporter AcrB. Nature.

[50] Zwama M, Yamaguchi A (2018). Molecular mechanisms of AcrB-mediated multidrug export. Res Microbiol.

[51] Zwama M, Nishino K (2021). Ever-adapting RND efflux pumps in gram-negative multidrug-resistant pathogens: A race against time. Antibiotics.

[52] Kobylka J, Kuth MS, Müller RT, Geertsma ER, Pos KM (2020). AcrB: A mean, keen, drug efflux machine. Ann N Y Acad Sci.

[53] Yang S, Lopez CR, Zechiedrich EL (2006). Quorum sensing and multidrug transporters in *Escherichia coli*. Proc Natl Acad Sci U S A.

[54] Rahmati S, Yang S, Davidson AL, Zechiedrich EL (2002). Control of the AcrAB multidrug efflux pump by quorum-sensing regulator SdiA. Mol Microbiol.

[55] Yamasaki S, Wang LY, Hirata T, Hayashi-Nishino M, Nishino K (2015). Multidrug efflux pumps contribute to *Escherichia coli* biofilm maintenance. Int J Antimicrob Agents.

[56] Bay DC, Stremick CA, Slipski CJ, Turner RJ (2017). Secondary multidrug efflux pump mutants alter *Escherichia coli* biofilm growth in the presence of cationic antimicrobial compounds. Res Microbiol.

[57] Aoki SK, Malinverni JC, Jacoby K, Thomas B, Pamma R (2008). Contact-dependent growth inhibition requires the essential outer membrane protein BamA (YaeT) as the receptor and the inner membrane transport protein AcrB. Mol Microbiol.

[58] Su C-C, Nikaido H, Yu EW (2007). Ligand-transporter interaction in the AcrB multidrug efflux pump determined by fluorescence polarization assay. FEBS Lett.

[59] Elkins CA, Mullis LB (2006). Mammalian steroid hormones are substrates for the major RND- and MFS-type tripartite multidrug efflux pumps of *Escherichia coli*. J Bacteriol.

[60] Zwama M, Yamasaki S, Nakashima R, Sakurai K, Nishino K (2018). Multiple entry pathways within the efflux transporter AcrB contribute to multidrug recognition. Nat Commun.

[61] Tam H-K, Malviya VN, Foong W-E, Herrmann A, Malloci G (2020). Binding and transport of carboxylated drugs by the multidrug transporter AcrB. J Mol Biol.

[62] Alav I, Bavro VN, Blair JMA (2022). A role for the periplasmic adaptor protein AcrA in vetting substrate access to the RND efflux transporter AcrB. Sci Rep.

[63] Murakami S, Nakashima R, Yamashita E, Matsumoto T, Yamaguchi A (2006). Crystal structures of a multidrug transporter reveal a functionally rotating mechanism. Nature.

[64] Nakashima R, Sakurai K, Yamasaki S, Nishino K, Yamaguchi A (2011). Structures of the multidrug exporter AcrB reveal a proximal multisite drug-binding pocket. Nature.

[65] Eicher T, Cha H, Seeger MA, Brandstätter L, El-Delik J (2012). Transport of drugs by the multidrug transporter AcrB involves an access and a deep binding pocket that are separated by a switch-loop. Proc Natl Acad Sci U S A.

[66] Li XZ, Nikaido H (2004). Efflux-mediated drug resistance in bacteria. Drugs.

[67] Grkovic S, Brown MH, Skurray RA (2002). Regulation of bacterial drug export systems. Microbiol Mol Biol Rev.

[68] Rosenberg EY, Bertenthal D, Nilles ML, Bertrand KP, Nikaido H (2003). Bile salts and fatty acids induce the expression of *Escherichia coli* AcrAB multidrug efflux pump through their interaction with Rob regulatory protein. Mol Microbiol.

[69] El Meouche I, Dunlop MJ (2018). Heterogeneity in efflux pump expression predisposes antibiotic-resistant cells to mutation. Science.

[70] Bleuel C, Grosse C, Taudte N, Scherer J, Wesenberg D (2005). TolC is involved in enterobactin efflux across the outer membrane of *Escherichia coli*. J Bacteriol.

[71] Deininger KNW, Horikawa A, Kitko RD, Tatsumi R, Rosner JL (2011). A requirement of TolC and MDR efflux pumps for acid adaptation and gadAB induction in *Escherichia coli*. PLoS One.

[72] Matsumura K, Furukawa S, Ogihara H, Morinaga Y (2011). Roles of multidrug efflux pumps on the biofilm formation of *Escherichia coli* K-12. Biocontrol Sci.

[73] Kobayashi N, Tamura N, van Veen HW, Yamaguchi A, Murakami S (2014). β-Lactam selectivity of multidrug transporters AcrB and AcrD resides in the proximal binding pocket. J Biol Chem.

[74] Zhang Z, Morgan CE, Cui M, Yu EW (2023). Cryo-EM structures of AcrD illuminate a mechanism for capturing aminoglycosides from its central cavity. mBio.

[75] Hirakawa H, Inazumi Y, Masaki T, Hirata T, Yamaguchi A (2005). Indole induces the expression of multidrug exporter genes in *Escherichia coli*. Mol Microbiol.

[76] Nishino K, Yamasaki S, Hayashi-Nishino M, Yamaguchi A (2010). Effect of NlpE overproduction on multidrug resistance in *Escherichia coli*. Antimicrob Agents Chemother.

[77] Lau SY, Zgurskaya HI (2005). Cell division defects in *Escherichia coli* deficient in the multidrug efflux transporter AcrEF-TolC. J Bacteriol.

[78] Wei Y, Lee J-M, Smulski DR, LaRossa RA (2001). Global impact of sdiA amplification revealed by comprehensive gene expression profiling of *Escherichia coli*. J Bacteriol.

[79] Nishino K, Yamaguchi A (2004). Role of histone-like protein H-NS in multidrug resistance of *Escherichia coli*. J Bacteriol.

[80] Kobayashi A, Hirakawa H, Hirata T, Nishino K, Yamaguchi A (2006). Growth phase-dependent expression of drug exporters in *Escherichia coli* and Its contribution to drug tolerance. J Bacteriol.

[81] Hirakawa H, Inazumi Y, Senda Y, Kobayashi A, Hirata T (2006). N-acetyl-d-glucosamine induces the expression of multidrug exporter genes, mdtEF, via catabolite activation in *Escherichia coli*. J Bacteriol.

[82] Zhang Y, Xiao M, Horiyama T, Zhang Y, Li X (2011). The multidrug efflux pump MdtEF protects against nitrosative damage during the anaerobic respiration in *Escherichia coli*. J Biol Chem.

[83] Hirakawa H, Nishino K, Yamada J, Hirata T, Yamaguchi A (2003). Beta-lactam resistance modulated by the overexpression of response regulators of two-component signal transduction systems in *Escherichia coli*. J Antimicrob Chemother.

[84] Masuda N, Church GM (2002). *Escherichia coli* gene expression responsive to levels of the response regulator EvgA. J Bacteriol.

[85] Nishino K, Yamasaki S, Hayashi-Nishino M, Yamaguchi A (2011). Effect of overexpression of small non-coding DsrA RNA on multidrug efflux in *Escherichia coli*. J Antimicrob Chemother.

[86] Nagakubo S, Nishino K, Hirata T, Yamaguchi A (2002). The putative response regulator BaeR stimulates multidrug resistance of *Escherichia coli* via a novel multidrug exporter system, MdtABC. J Bacteriol.

[87] Wang D, Fierke CA (2013). The BaeSR regulon is involved in defense against zinc toxicity in *Escherichia coli*. Metallomics.

[88] Leblanc SKD, Oates CW, Raivio TL (2011). Characterization of the induction and cellular role of the BaeSR two-component envelope stress response of *Escherichia coli*. J Bacteriol.

[89] Long F, Su C-C, Lei H-T, Bolla JR, Do SV (2012). Structure and mechanism of the tripartite CusCBA heavy-metal efflux complex. Philos Trans R Soc Lond B Biol Sci.

[90] Kim E-H, Nies DH, McEvoy MM, Rensing C (2011). Switch or funnel: How RND-type transport systems control periplasmic metal homeostasis. J Bacteriol.

[91] Long F, Su C-C, Zimmermann MT, Boyken SE, Rajashankar KR (2010). Crystal structures of the CusA efflux pump suggest methionine-mediated metal transport. Nature.

[92] Poole K (2007). Efflux pumps as antimicrobial resistance mechanisms. Ann Med.

[93] Yang L, Chen L, Shen L, Surette M, Duan K (2011). Inactivation of MuxABC-OpmB transporter system in *Pseudomonas aeruginosa* leads to increased ampicillin and carbenicillin resistance and decreased virulence. J Microbiol.

[94] Langendonk RF, Neill DR, Fothergill JL (2021). The building blocks of antimicrobial resistance in *Pseudomonas aeruginosa*: Implications for current resistance-breaking therapies. Front Cell Infect Microbiol.

[95] Poole K, Krebes K, McNally C, Neshat S (1993). Multiple antibiotic resistance in *Pseudomonas aeruginosa*: evidence for involvement of an efflux operon. J Bacteriol.

[96] Pearson JP, Van Delden C, Iglewski BH (1999). Active efflux and diffusion are involved in transport of *Pseudomonas aeruginosa* cell-to-cell signals. J Bacteriol.

[97] Köhler T, van Delden C, Curty LK, Hamzehpour MM, Pechere JC (2001). Overexpression of the MexEF-OprN multidrug efflux system affects cell-to-cell signaling in *Pseudomonas aeruginosa*. J Bacteriol.

[98] Aendekerk S, Ghysels B, Cornelis P, Baysse C (2002). Characterization of a new efflux pump, MexGHI-OpmD, from *Pseudomonas aeruginosa* that confers resistance to vanadium. Microbiology.

[99] Sánchez P, Linares JF, Ruiz-Díez B, Campanario E, Navas A (2002). Fitness of in vitro selected *Pseudomonas aeruginosa* nalB and nfxB multidrug resistant mutants. J Antimicrob Chemother.

[100] Evans K, Passador L, Srikumar R, Tsang E, Nezezon J (1998). Influence of the MexAB-OprM multidrug efflux system on quorum sensing in *Pseudomonas aeruginosa*. J Bacteriol.

[101] Lutter EI, Purighalla S, Duong J, Storey DG (2012). Lethality and cooperation of *Pseudomonas aeruginosa* quorum-sensing mutants in drosophila melanogaster infection models. Microbiology.

[102] Moore JD, Gerdt JP, Eibergen NR, Blackwell HE (2014). Active efflux influences the potency of quorum sensing inhibitors in *Pseudomonas aeruginosa*. Chembiochem.

[103] Sawada I, Maseda H, Nakae T, Uchiyama H, Nomura N (2004). A quorum-sensing autoinducer enhances the mexAB-oprM efflux-pump expression without the MexR-mediated regulation in *Pseudomonas aeruginosa*. Microbiol Immunol.

[104] Minagawa S, Inami H, Kato T, Sawada S, Yasuki T (2012). RND type efflux pump system MexAB-OprM of *Pseudomonas aeruginosa* selects bacterial languages, 3-oxo-acyl-homoserine lactones, for cell-to-cell communication. BMC Microbiol.

[105] Olivares Pacheco J, Alvarez-Ortega C, Alcalde Rico M, Martínez JL (2017). Metabolic compensation of fitness costs is a general outcome for antibiotic-resistant *Pseudomonas aeruginosa* mutants overexpressing efflux pumps. mBio.

[106] Hirakata Y, Kondo A, Hoshino K, Yano H, Arai K (2009). Efflux pump inhibitors reduce the invasiveness of *Pseudomonas aeruginosa*. Int J Antimicrob Agents.

[107] Hirakata Y, Srikumar R, Poole K, Gotoh N, Suematsu T (2002). Multidrug efflux systems play an important role in the invasiveness of *Pseudomonas aeruginosa*. J Exp Med.

[108] Kondo A, Hirakata Y, Gotoh N, Fukushima K, Yanagihara K (2006). Quorum sensing system lactones do not increase invasiveness of a MexAB-OprM efflux mutant but do play a partial role in *Pseudomonas aeruginosa* invasion. Microbiol Immunol.

[109] De Kievit TR, Parkins MD, Gillis RJ, Srikumar R, Ceri H (2001). Multidrug efflux pumps: Expression patterns and contribution to antibiotic resistance in *Pseudomonas aeruginosa* biofilms. Antimicrob Agents Chemother.

[110] Liao J, Schurr MJ, Sauer K (2013). The MerR-like regulator BrlR confers biofilm tolerance by activating multidrug efflux pumps in *Pseudomonas aeruginosa* biofilms. J Bacteriol.

[111] Linares JF, López JA, Camafeita E, Albar JP, Rojo F (2005). Overexpression of the multidrug efflux pumps MexCD-OprJ and MexEF-OprN is associated with a reduction of type III secretion in *Pseudomonas aeruginosa*. J Bacteriol.

[112] Stickland HG, Davenport PW, Lilley KS, Griffin JL, Welch M (2010). Mutation of nfxB causes global changes in the physiology and metabolism of *Pseudomonas aeruginosa*. J Proteome Res.

[113] Martínez-Ramos I, Mulet X, Moyá B, Barbier M, Oliver A (2014). Overexpression of MexCD-OprJ reduces *Pseudomonas aeruginosa* virulence by increasing its susceptibility to complement-mediated killing. Antimicrob Agents Chemother.

[114] Jeannot K, Elsen S, Köhler T, Attree I, van Delden C (2008). Resistance and virulence of *Pseudomonas aeruginosa* clinical strains overproducing the MexCD-OprJ efflux pump. Antimicrob Agents Chemother.

[115] Zaborskyte G, Andersen JB, Kragh KN, Ciofu O (2017). Real-time monitoring of nfxB mutant occurrence and dynamics in *Pseudomonas aeruginosa* biofilm exposed to subinhibitory concentrations of ciprofloxacin. Antimicrob Agents Chemother.

[116] Mandsberg LF, Ciofu O, Kirkby N, Christiansen LE, Poulsen HE (2009). Antibiotic resistance in *Pseudomonas aeruginosa* strains with increased mutation frequency due to inactivation of the DNA oxidative repair system. Antimicrob Agents Chemother.

[117] Mandsberg LF, Maciá MD, Bergmann KR, Christiansen LE, Alhede M (2011). Development of antibiotic resistance and up-regulation of the antimutator gene pfpI in mutator *Pseudomonas aeruginosa* due to inactivation of two DNA oxidative repair genes (mutY, mutM). FEMS Microbiol Lett.

[118] Strempel N, Neidig A, Nusser M, Geffers R, Vieillard J (2013). Human host defense peptide LL-37 stimulates virulence factor production and adaptive resistance in *Pseudomonas aeruginosa*. PLoS One.

[119] Gillis RJ, White KG, Choi K-H, Wagner VE, Schweizer HP (2005). Molecular basis of azithromycin-resistant *Pseudomonas aeruginosa* biofilms. Antimicrob Agents Chemother.

[120] Olivares J, Alvarez-Ortega C, Linares JF, Rojo F, Köhler T (2012). Overproduction of the multidrug efflux pump MexEF-OprN does not impair *Pseudomonas aeruginosa* fitness in competition tests, but produces specific changes in bacterial regulatory networks. Environ Microbiol.

[121] Lamarche MG, Déziel E (2011). MexEF-OprN efflux pump exports the *Pseudomonas* quinolone signal (PQS) precursor HHQ (4-hydroxy-2-heptylquinoline). PLoS One.

[122] Oshri RD, Zrihen KS, Shner I, Omer Bendori S, Eldar A (2018). Selection for increased quorum-sensing cooperation in *Pseudomonas aeruginosa* through the shut-down of a drug resistance pump. ISME J.

[123] Tian Z-X, Mac Aogáin M, O’Connor HF, Fargier E, Mooij MJ (2009). MexT modulates virulence determinants in *Pseudomonas aeruginosa* independent of the MexEF-OprN efflux pump. Microb Pathog.

[124] Jin Y, Yang H, Qiao M, Jin S (2011). MexT regulates the type III secretion system through MexS and PtrC in *Pseudomonas aeruginosa*. J Bacteriol.

[125] Wang D, Seeve C, Pierson LS, Pierson EA (2013). Transcriptome profiling reveals links between ParS/ParR, MexEF-OprN, and quorum sensing in the regulation of adaptation and virulence in *Pseudomonas aeruginosa*. BMC Genomics.

[126] Balasubramanian D, Schneper L, Merighi M, Smith R, Narasimhan G (2012). The regulatory repertoire of *Pseudomonas aeruginosa* AmpC ß-lactamase regulator AmpR includes virulence genes. PLoS One.

[127] Juarez P, Jeannot K, Plésiat P, Llanes C (2017). Toxic electrophiles induce expression of the multidrug efflux pump MexEF-OprN in *Pseudomonas aeruginosa* through a novel transcriptional regulator, CmrA. Antimicrob Agents Chemother.

[128] Kumar A, Schweizer HP, Van Melderen L (2011). Evidence of MexT-independent overexpression of MexEF-OprN multidrug efflux pump of *Pseudomonas aeruginosa* in presence of metabolic stress. PLoS One.

[129] Fargier E, Mac Aogáin M, Mooij MJ, Woods DF, Morrissey JP (2012). MexT functions as a redox-responsive regulator modulating disulfide stress resistance in *Pseudomonas aeruginosa*. J Bacteriol.

[130] Schaible B, Taylor CT, Schaffer K (2012). Hypoxia increases antibiotic resistance in *Pseudomonas aeruginosa* through altering the composition of multidrug efflux pumps. Antimicrob Agents Chemother.

[131] Fetar H, Gilmour C, Klinoski R, Daigle DM, Dean CR (2011). mexEF-oprN multidrug efflux operon of *Pseudomonas aeruginosa*: regulation by the MexT activator in response to nitrosative stress and chloramphenicol. Antimicrob Agents Chemother.

[132] Caughlan RE, Sriram S, Daigle DM, Woods AL, Buco J (2009). Fmt bypass in *Pseudomonas aeruginosa* causes induction of MexXY efflux pump expression. Antimicrob Agents Chemother.

[133] Jeannot K, Sobel ML, El Garch F, Poole K, Plésiat P (2005). Induction of the MexXY efflux pump in *Pseudomonas aeruginosa* is dependent on drug-ribosome interaction. J Bacteriol.

[134] Hay T, Fraud S, Lau CH-F, Gilmour C, Poole K (2013). Antibiotic inducibility of the mexXY multidrug efflux operon of *Pseudomonas aeruginosa*: involvement of the MexZ anti-repressor ArmZ. PLoS One.

[135] Muller C, Plésiat P, Jeannot K (2011). A two-component regulatory system interconnects resistance to polymyxins, aminoglycosides, fluoroquinolones, and β-lactams in *Pseudomonas aeruginosa*. Antimicrob Agents Chemother.

[136] Poole K, Gilmour C, Farha MA, Mullen E, Lau CH-F (2016). Potentiation of aminoglycoside activity in *Pseudomonas aeruginosa* by targeting the AmgRS envelope stress-responsive two-component system. Antimicrob Agents Chemother.

[137] Lau CHF, Krahn T, Gilmour C, Mullen E, Poole K (2015). AmgRS-mediated envelope stress-inducible expression of the mexXY multidrug efflux operon of *Pseudomonas aeruginosa*. MicrobiologyOpen.

[138] Shi J, Jin Y, Bian T, Li K, Sun Z (2015). SuhB is a novel ribosome associated protein that regulates expression of MexXY by modulating ribosome stalling in *Pseudomonas aeruginosa*. Mol Microbiol.

[139] Fraud S, Poole K (2011). Oxidative stress induction of the MexXY multidrug efflux genes and promotion of aminoglycoside resistance development in *Pseudomonas aeruginosa*. Antimicrob Agents Chemother.

[140] Aendekerk S, Diggle SP, Song Z, Høiby N, Cornelis P (2005). The MexGHI-OpmD multidrug efflux pump controls growth, antibiotic susceptibility and virulence in *Pseudomonas aeruginosa* via 4-quinolone-dependent cell-to-cell communication. Microbiology.

[141] Dietrich LEP, Price-Whelan A, Petersen A, Whiteley M, Newman DK (2006). The phenazine pyocyanin is a terminal signalling factor in the quorum sensing network of *Pseudomonas aeruginosa*. Mol Microbiol.

[142] Sakhtah H, Koyama L, Zhang Y, Morales DK, Fields BL (2016). The *Pseudomonas aeruginosa* efflux pump MexGHI-OpmD transports a natural phenazine that controls gene expression and biofilm development. Proc Natl Acad Sci U S A.

[143] Sporer AJ, Beierschmitt C, Bendebury A, Zink KE, Price-Whelan A (2018). *Pseudomonas aeruginosa* PumA acts on an endogenous phenazine to promote self-resistance. Microbiology.

[144] Perron K, Caille O, Rossier C, Van Delden C, Dumas J-L (2004). CzcR-CzcS, a two-component system involved in heavy metal and carbapenem resistance in *Pseudomonas aeruginosa*. J Biol Chem.

[145] Caille O, Rossier C, Perron K (2007). A copper-activated two-component system interacts with zinc and imipenem resistance in *Pseudomonas aeruginosa*. J Bacteriol.

[146] Lassmann T (2019). Kalign 3: Multiple sequence alignment of large data sets. Bioinformatics.

[147] Letunic I, Bork P (2021). Interactive tree of life (iTOL) v5: An online tool for phylogenetic tree display and annotation. Nucleic Acids Res.

[148] Barchiesi J, Castelli ME, Soncini FC, Véscovi EG (2008). MgtA expression is induced by rob overexpression and mediates a *Salmonella enterica* resistance phenotype. J Bacteriol.

[149] Fàbrega A, du Merle L, Le Bouguénec C, Jiménez de Anta MT, Vila J (2009). Repression of invasion genes and decreased invasion in a high-level fluoroquinolone-resistant *Salmonella* typhimurium mutant. PLoS One.

[150] Lawler AJ, Ricci V, Busby SJW, Piddock LJV (2013). Genetic inactivation of acrAB or inhibition of efflux induces expression of ramA. J Antimicrob Chemother.

[151] Yamasaki S, Nagasawa S, Fukushima A, Hayashi-Nishino M, Nishino K (2013). Cooperation of the multidrug efflux pump and lipopolysaccharides in the intrinsic antibiotic resistance of *Salmonella enterica* serovar Typhimurium. J Antimicrob Chemother.

[152] Rensch U, Nishino K, Klein G, Kehrenberg C (2014). *Salmonella enterica* serovar typhimurium multidrug efflux pumps emrAB and AcrEF support the major efflux system AcrAB in decreased susceptibility to triclosan. Int J Antimicrob Agents.

[153] Yamasaki S, Fujioka T, Hayashi K, Yamasaki S, Hayashi-Nishino M (2016). Phenotype microarray analysis of the drug efflux systems in *Salmonella enterica* serovar Typhimurium. J Infect Chemother.

[154] Li L, Yang Y-R, Liao X-P, Lei C-Y, Sun J (2013). Development of ceftriaxone resistance affects the virulence properties of *Salmonella enterica* serotype Typhimurium strains. Foodborne Pathog Dis.

[155] Masuda N, Sakagawa E, Ohya S, Gotoh N, Tsujimoto H (2000). Substrate specificities of mexAB-OprM, mexCD-OprJ, and mexXY-OprM efflux pumps. Antimicrob Agents Chemother.

[156] Mima T, Kohira N, Li Y, Sekiya H, Ogawa W (2009). Gene cloning and characteristics of the RND-type multidrug efflux pump MuxABC-OpmB possessing two RND components in *Pseudomonas aeruginosa*. Microbiology.

[157] Poole K (2001). Multidrug efflux pumps and antimicrobial resistance in *Pseudomonas aeruginosa* and related organisms. J Mol Microbiol Biotechnol.

[158] Fraud S, Campigotto AJ, Chen Z, Poole K (2008). MexCD-OprJ multidrug efflux system of *Pseudomonas aeruginosa*: involvement in chlorhexidine resistance and induction by membrane-damaging agents dependent upon the AlgU stress response sigma factor. Antimicrob Agents Chemother.

[159] Wolloscheck D, Krishnamoorthy G, Nguyen J, Zgurskaya HI (2018). Kinetic control of quorum sensing in *Pseudomonas aeruginosa* by multidrug efflux pumps. ACS Infect Dis.

[160] Sekiya H, Mima T, Morita Y, Kuroda T, Mizushima T (2003). Functional cloning and characterization of a multidrug efflux pump, mexHI-opmD, from a *Pseudomonas aeruginosa* mutant. Antimicrob Agents Chemother.

[161] Mima T, Sekiya H, Mizushima T, Kuroda T, Tsuchiya T (2005). Gene cloning and properties of the RND-type multidrug efflux pumps MexPQ-OpmE and MexMN-OprM from *Pseudomonas aeruginosa*. Microbiol Immunol.

[162] Li Y, Mima T, Komori Y, Morita Y, Kuroda T (2003). A new member of the tripartite multidrug efflux pumps, MexVW-OprM, in *Pseudomonas aeruginosa*. J Antimicrob Chemother.

[163] Chuanchuen R, Murata T, Gotoh N, Schweizer HP (2005). Substrate-dependent utilization of OprM or OpmH by the *Pseudomonas aeruginosa* MexJK efflux pump. Antimicrob Agents Chemother.

[164] Mima T, Joshi S, Gomez-Escalada M, Schweizer HP (2007). Identification and characterization of TriABC-OpmH, a triclosan efflux pump of *Pseudomonas aeruginosa* requiring two membrane fusion proteins. J Bacteriol.

[165] Wang Z, Fan G, Hryc CF, Blaza JN, Serysheva II (2017). An allosteric transport mechanism for the AcrAB-TolC multidrug efflux pump. Elife.

