# Supplementary material for: Drug resistance and physiological roles of RND multidrug efflux pumps in Salmonella enterica, Escherichia coli and Pseudomonas aeruginosa
Source: Microbiology (Reading). 2023 Jun 15;169(6):001322. doi: 10.1099/mic.0.001322 (PMC10333786; doi:10.1099/mic.0.001322)
Supplement: Supplementary material 1 [file mic-169-1322-s001.pdf]

Supplementary file

Drug resistance and physiological roles of RND multidrug efflux pumps in *Salmonella enterica*, *Escherichia coli,* and *Pseudomonas aeruginosa*

Seiji Yamasaki[1,2,3†], Martijn Zwama[1†], Tomohiro Yoneda[1,2†], Mitsuko Hayashi-Nishino[1,2], and Kunihiko Nishino[1,2,4*]

[1]*SANKEN (The Institute of Scientific and Industrial Research), Osaka University, 8-1 Mihogaoka, Ibaraki, Osaka 567-0047, Japan*
[2]*Graduate School of Pharmaceutical Sciences, Osaka University, 1-6 Yamadaoka, Suita, Osaka 565-0871, Japan*
[3]*Institute for Advanced Co-Creation Studies, Osaka University, 1-1 Yamadaoka, Suita, Osaka 565-0871, Japan*
[4]*Center for Infectious Disease Education and Research, 2-8 Yamadaoka, Osaka University, Suita, Osaka 565-0871, Japan*
[†]These authors contributed equally to this study

[*]Corresponding author
Mailing address: Institute of Scientific and Industrial Research, Osaka University, 8-1 Mihogaoka, Ibaraki, Osaka 567-0047, Japan
Tel: +81-6-6879-8548; Fax: +81-6-6879-8549; E-mail: nishino@sanken.osaka-u.ac.jp

Table S1. **Overview of the amino acid sequences of all RND-type transporters compared in this review.**

#	Protein (RND) name	Ref (locus/ncbi)	Sequence (aa)
1	MexB_Pa	PA0426 (mexB)	MSKFFIDRPIFAWVIALVIMLAGGLSILSLPVNQYPAIAPPAIAVQVSYPGASAETVQDTVVQVIEQQMNGIDNLRYISSESNSDGSMTITVTFEQGTDPDIAQVQVQNKLQLATPLLPQEVQRQGIR<br>VTKAVKNFLMVVGVVSTDGSMTKEDLSNYIVSNIQDPLSRTKGVGDFQVFGSQYSMRIWLDPAKLNSYQLTPGDVSSAIQAQNVQISSGQLGGLPAVKGQQLNATIIGKTRLQTAEQFENILLKVN<br>PDGSQVRLKDVADVGLGGQDYSINAQFNGSPASGIAIKLATGANALDTAKAIRQTIANLEPFMPQGMKVVYPYDTTPVVSASIHEVVKTLGEAILLVFLVMYLFLQNFRATLIPTIAVPVVLLGTFGVL<br>AAFGFSINTLTMFGMVLAIGLLVDDAIVVVENVERVMAEEGLSPREAARKSMGQIQGALVGIAMVLSAVFLPMAFFGGSTGVIYRQFSITIVSAMALSVIVALILTPALCATMLKPIEKGDHGEHKGG<br>FFGWFNRMFLSTTHGYERGVASILKHRAPYLLIYVVIVAGMIWMFTRIPTAFLPDEDQGVLFAQVQTPPGSSAERTQVVVDSMREYLLEKESSSVSSVFTVTGFNFAGRGQSSGMAFIMLKPWEE<br>RPGGENSVFELAKRAQMHFFSFKDAMVFAFAPPSVLELGNATGFDLFLQDQAGVGHEVLLQARNKFLMLAAQNPALQRVRPNGMSDEPQYKLEIDDEKASALGVSLADINSTVSIAWGSSYVND<br>FIDRGRVKRVYLQGRPDARMNPDDLSKWYVRNDKGEMVPFNAFATGKWEYGSPKLERYNGVPAMEILGEPAPGLSSGDAMAAVEEIVKQLPKGVGYSWTGLSYEERLSGSQAPALYALSLLVVF<br>LCLAALYESWSIPFSVMLVVPLGVIGALLATSMRGLSNDVFFQVGLLTTIGLSAKNAILIVEFAKELHEQGKGIVEAAIEACRMRLRPIVMTSLAFILGVVPLAISTGAGSGSQHAIGTGVIGGMVTATV<br>LAIFWVPLFYVAVSTLFKDEASKQQASVEKGQ
2	MexD_Pa	PA4598 (mexD)	MSEFFIKRPNFAWVVALFISLAGLLVISKLPVAQYPNVAPPQITITATYPGASAKVLVDSVTSVLEESLNGAKGLLYFESTNNSNGTAEIVVTFEPGTDPDLAQVDVQNRLKKAEARMPQAVLTQGLQ<br>VEQTSAGFLLIYALSYKEGAQRSDTTALGDYAARNINNELRRLPGVGKLQFFSSEAAMRVWIDPQKLVGFGLSIDDVSNAIRGQNVQVPAGAFGSAPGSSAQELTATLAVKGTLDDPQEFGQVVLR<br>ANEDGSLVRLADVARLELGKESYNISSRLNGTPTVGGAIQLSPGANAIQTATLVKQRLAELSAFFPEDMQYSVPYDTSRFVDVAIEKVIHTLIEAMVLVFLVMFLFLQNVRYTLIPSIVVPVCLLGTLMV<br>MYLLGFSVNMMTMFGMVLAIGILVDDAIVVVENVERIMAEEGISPAEATVKAMKQVSGAIVGITLVLSAVFLPLAFMAGSVGVIYQQFSVSLAVSILFSGFLALTFTPALCATLLKPIPEGHHEKRGFF<br>GAFNRGFARVTERYSLLNSKLVARAGRFMLVYAGLVAMLGYFYLRLPEAFVPAEDLGYMVVDVQLPPGASRVRTDATGEELERFLKSREAVASVFLISGFSFSGQGDNAALAFPTFKDWSERGAE<br>QSAAAEIAALNEHFALPDDGTVMAVSPPPINGLGNSGGFALRLMDRSGVGREALLQARDTLLGEIQTNPKFLYAMMEGLAEAPQLRLLIDREKARALGVSFETISGTLSAAFGSEVINDFTNAGRQ<br>QRVVIQAEQGNRMTPESVLELYVPNAAGNLVPLSAFVSVKWEEGPVQLVRYNGYPSIRIVGDAAPGFSTGEAMAEMERLASQLPAGIGYEWTGLSYQEKVSAGQATSLFALAILVVFLLLVALYES<br>WSIPLSVMLIVPIGAIGAVLAVMVSGMSNDVYFKVGLITIIGLSAKNAILIVEFAKELWEQGHSLRDAAIEAARLRFRPIIMTSMAFILGVIPLALASGAGAASQRAIGTGVIGGMLSATFLGVLFVPICF<br>VWLLSLLRSKPAPIEQAASAGE
3	MexF_Pa	PA2494 (mexF)	MNFSQFFIQRPIFAAVLSLLILIGGAISLFQLPISEYPEVVPPTVVVRANFPGANPKVIGETVASPLEQAITGVENMLYMSSQSTSDGKLTLTITFALGTDLDNAQVQVQNRVTRTEPKLPEEVTRLGITV<br>DKASPDLTMVVHLTSPDNRYDMLYLSNYAVLNVKDELARLDGVGDVQLFGLGDYSLRVWLDPNKVASRNLTATDVVNAIREQNRQVAAGTLGAPPAPSDTSFQLSINTQGRLVTEEEFENIIIRAG<br>ANGEITRLRDIARVELGSNQYALRSLLNNKPAVAIPIFQRPGSNAIEISNLVREKMAELKHSFPQGMDYSIVYDPTIFVRGSIEAVVHTLFEALVLVVLVVILFLQTWRASIIPLAAVPVSLIGTFAVMHM<br>LGFSLNALSLFGLVLAIGIVVDDAIVVVENVERNIGLGLKPVEATKRAMREVTGPIIATALVLCAVFIPTAFISGLTGQFYRQFALTIAISTVISAFNSLTLSPALAAVLLKGHHEPKDRFSVFLDKLLGSWLF<br>RPFNRFFDRASHGYVGTVNRVLRGSSIALLVYGGLMVLTYFGFSSTPTGFVPQQDKQYLVAFAQLPDAASLDRTEAVIKQMSEIALAQPGVADSVAFPGLSINGFTNSPNSGIVFTPLKPFDERKDPS<br>QSAGAIAAALNAKYADIQDAYIAIFPPPPVQGLGTIGGFRLQIEDRGNQGYEELFKQTQNIITKARALPELEPSSVFSSYQVNVPQIDADIDREKAKTHGVAISDIFDTLQVYLGSLYANDFNRFGRTYQ<br>VNVQAEQQFRLEPEQIGQLKVRNNLGEMVPLASFIKVSDTSGPDRVMHYNGFITAELNGAPAAGYSSGQAQAAIEKLLKEELPNGMTYEWTELTYQQILAGNTALFVFPLCVLLAFLVLAAQYESW<br>SLPLAVILIVPMTLLSAITGVILAGSDNNIFTQIGLIVLVGLACKNAILIVEFAKDKQEEGMDRVAAVLEACRLRLRPILMTSIAFIMGVVPLVISTGAGAEMRHAMGVAVFSGMIGVTFFGLLLTPVFYV<br>LIRRFVENREARRAANDKGLPEVHA
4	MexY_Pa	PA2018 (mexY)	MARFFIDRPVFAWVISLLIVLAGVLAIRFLPVAQYPDIAPPVVNVSASYPGASAKVVEEAVTAIIEREMNGAPGLLYTKATSSTGQASLTLTFRQGVNADLAAVEVQNRLKIVESRLPESVRRDGIYVEK<br>AADSIQLIVTLTSSSGRYDAMELGEIASSNVLQALRRVEGVGKVETWGAEYAMRIWPDPAKLTSMNLSASDLVNAVRRHNARLTVGDIGNLGVPDSAPISATVKVDDTLVTPEQFGEIPLRIRADGG<br>AIRLRDVARVEFGQSEYGFVSRVNQMTATGLAVKMAPGSNAVATAKRIRATLDELSRYFPEGVSYNIPYDTSAFVEISIRKVVSTLLEAMLLVFAVMYLFMQNFRATLIPTLVVPVALLGTFTVMLGL<br>GFSINVLTMFGMVLAIGILVDDAIIVVENVERLMAEEGLSPHDATVKAMRQISGAIVGITVVLVSVFVPMAFFSGAVGNIYRQFAVTLAVSIGFSAFLALSLTPALCATLLRPIDADHHEKRGFFGWFN<br>RAFLRLTGRYRNAVAGILARPIRWMLVYTLVIGVVALLFVRLPQAFLPEEDQGDFMIMVMQPEGTPMAETMANVGDVERYLAEHEPVAYAYAVGGFSLYGDGTSSAMIFATLKDWSERREASQH<br>VGAIVERINQRFAGLPNRTVYAMNSPPLPDLGSTSGFDFRLQDRGGVGYEALVKARDQLLARAAEDPRLANVMFAGQGEAPQIRLDIDRRKAETLGVSMDEINTTLAVMFGSDYIGDFMHGSQV<br>RKVVVQADGAKRLGIDDIGRLHVRNEQGEMVPLATFAKAAWTLGPPQLTRYNGYPSFNLEGQAAPGYSSGEAMQAMEQLMQGLPEGIAHEWSGQSFEERLSGAQAPALFALSVLIVFLALAALY<br>ESWSIPLAVILVVPLGVLGALLGVSLRGLPNDIYFKVGLITIIGLSAKNAILIIEVAKDHYQEGMSLLQATLEAARLRLRPIVMTSLAFGFGVVPLALSSGAGSGAQVAIGTGVLGGIVTATVLAVFLVPLF<br>FLVVGRLFRLRKAPRTGNSPQIPTEQA
5	MexK_Pa	PA3676 (mexK)	MSFNLSAWALQNRQIVLYLMILLGAVGALSYSKLGQSEDPPFTFKAMVVQTNWPGASAEEVARQVTERIEKKLMETGDYDRIVSFSRPGVSQVTFMAREDIHSSEIPELWYQIRKKISDIRATLPQSI<br>QGPFFNDEFGTTYGNIYALTGKGFDYAVMKDYADRLQLQLQRIRNVGKVELIGLQDEKIWIDLSNTKLATLGLPLAAVQKALEEQNAVASSGFFETASDRVQLRVSGRFDSVEEIRDFPIRVGDRTFR<br>IGDVAEVRRGFNDPPAPRMRFMGEDAIGLAVAMKPGGDILVLGKALETEFARLQQSLPAGLELRKVSDQPAAVRTGVGEFIRVLAEALVIVLLVSFFSLGLRTGLVVALSIPLVLAMTFAAMHYFGIG<br>LHKISLGALVLALGLLVDDAIIAVEMMAVKMEQGYDRLKAASFAWTSTAFPMLTGTLITAAGFLPIATAQSGTGEYTRSLFQVVTIALVVSWFAAVVFVPYLGAKLLPDLARLHAQKHGGSADGYDPY<br>ATAFYQRFRRLVEWCVRYRKTVIVLTLAAFVGALLLFRLVPQQFFPPSARLELLLDIKLAEGASLRSTGEEVQRLEKMLQGHDGIDNYVAYVGTGSPRFYLPLDQQLPAASFAQVVVLAKDLESREALR<br>KWLIERMNEDFPHLRSRISRLENGPPVGYPVQFRVSGEDIPQVRELARKVADKMRENPHVVNVHLDWEEPSKVVYLSIDQERARALGVSTASLSQFLQSALTGSHVSFFREDNELIEILLRGTEQERR<br>DLSLLPSLAVPTENGRSVALSQIATLEYGFEEGIIWHRNRLPTVTVRADIYDDSLPATLVAQIAPTLEPIRAELPDGYLLEVGGTVEDAAKGQSSVNAGVPLFIVVVLSLLMVQLRSFSRMAMVFLTAPL<br>GLIGVTLFLLLFRQPFGFVAMLGTIALAGMIMRNSVILVDQIEQDISHGLDRWHAIIEATVRRFRPIVLTALAAVLAMIPLSRSVFFGPMAVAIMGGLIVATVLTLLFLPALYAAWFRVKKDEARA
6	MexN_Pa	WP_003147398.1 / PA1438	MTPRAGISGWCVRHPIATALLTLASLLLGLLAFLRLGVAPLPEADFPTIQINALLPGGSPETMASSVATPLEVQFSAIPGITEMTSSSALGTTTLTLQFSLDKSIDVAAQEVQAAINAAAGRLPVDMPNL<br>PTWRKVNPADSPIMILRVNSEMMPLIELSDYAETILARQLSQVNGVGQIFVVGQQRPAIRIQAQPEKLAAYQLTLADLRQSLQSASVNLAKGALYGEGRVSTLAANDQLFNASDYDDLVVAYRQGA<br>PVFLKDVARIVSAPEDDYVQAWPNGVPGVALVILRQPGANIVDTADAIQAALPRLREMLPATIEVDVLNDRTRTIRSSLHEVELTLLLTIGLVVLVMGLFLRQLSATLIVATVLAVSLSASFAAMYVLGF<br>TLNNLTLVALIIAVGFIVDDAIVVVENIHRHLEAGASKVEAALKGAAEIGFTVISISFSLIAAFIPLLFMGGIVGRLFREFAVSVTVAILISVVASLTLAPMLASRFMPALRHAEAPRKGFAEWLTGGYERG<br>LRWALGHQRLMLVGFAFTVLVAVAGYVGIPKGFFPLQDTAFVFGTSQAAEDISYDDMVAKHRQLAEIIASDPAVQSYNHAVGVTGGSQSLANGRFWIVLKDRGERDVSVGEFIDRLRPQLAKVPG<br>IMLYLRAAQDINLSSGPSRTQYQYALRSSDSTQLALWAQRLTERLKQVPGLMDVSNDLQVGASVTALDIDRVAAARFGLSAEDVSQTLYDAFGQRQVGEYQTEVNQYKVVLELDARQRGRAESLD<br>WFYLRSPLSGEMVPLSAIAKVAAPRSGPLQINHNGMFPAVNLSFNLAAGVSLGEAVQAVQRAQEEIGMPSTIIGVFQGAAQAFQSSLASQPLLILAALIAVYIILGVLYESFVHPLTILSTLPSAGIGAV<br>FLLWAWGQDFSIMALIGIVLLIGIVKKNGILMVDFAIVAQREQGMSAEQAIYQACLTRFRPIMMTTLAALLGAIPLMIGFGTGSELRQPLGIAVVGGLLVSQVLTLFSTPVVYLALERLFHRRGTTTSD<br>GGTAGATAT
7	MexI_Pa	PA4207 (mexI)	MTFTDLFVRRPVLALVVSTLILLLGLFSLGKLPIRQYPLLESSTITVTTEYPGASADLMQGFVTQPIAQAVSSVEGIDYLSSTSVQGRSVVTIRMLLNRDSTQAMTETMAKVNSVRYKLPERAYDSVIER<br>SSGETTAVAYVGFSSKTLPIPALTDYLSRVVEPMFSSIDGVAKVQTFGGQRLAMRLWLDADRLAGRGLTASDVAEAIRRNNYQAAPGMVKGQYVLSNVRVNTDLTNVDDFREMVIRNDGNGLV<br>RLRDVGTVELGAAATETSALMDGDPAVHLGLFPTPTGNPLVIVDGIRKLLPEIQKTLPPDVRVDLAYETSRFIQASIDEVVRTLVEALLIVVLVIYLCLGSLRSVLIPVATIPLSMLGAAALMLAFGFSVNLL<br>TLLAMVLAIGLVVDDAIVVVENVHRHIEEGKSPVAAALIGAREVAGPVIAMTITLAAVYTPIGLMGGLTGALFREFALTLAGAVIVSGVVALTLSPVMSSLLLQAHQNEGRMGRAAEWFFGGLTRRY<br>GQVLEFSLGHRWLTGGLALLVCISLPLLYSMPKRELAPTEDQAAVLTAIKAPQHANLDYVELFARKLDQVYTSIPETVSTWIINGTDGPAASFGGINLAAWEKRERDASAIQSELQGKVGDVEGSSIFA<br>FQLAALPGSTGGLPVQMVLRSPQDYPVLYRTMEEIKQKARQSGLFVVVDSDLDYNNPVVQVRIDRAKANSLGIRMQDIGESLAVLVGENYVNRFGMEGRSYDVIPQSLRDQRFTPQALARQFVRT<br>QDGNLVPLSTVVRVALQVEPNKLIQFDQQNAATLQAIPAPGVSMGQAVAFLDDVARGLPAGFSHDWQSDSRQYTQEGNTLVFAFLAALVVIYLVLAAQYESLADPLIIILITVPLSICGALLPLALGYAT<br>MNIYTQIGLVTLIGLISKHGILMVEFANELQLHERLDRRAAILRAAQIRLRPVLMTTAAMVFGLVPLLFASGAGAASRFGLGVVIVSGMLVGTLFTLFVLPTVYTLLARNHAEVDKSPRSRQLAEADLLV<br>NKA
8	MexQ_Pa	PA3522 (mexQ)	MTFPRFFIDRPIFAIVLSVLMMIGGIVSFFQLPLSEYPAVTPPTVQVTTAYPGANPDVIAQTVATPLEQAITGVEGMLYMSSQSATDGRMILTITFDQHIDPDMAQIQVQNRVSRVLSRLPDEVQRQG<br>VVTQKTSPDILMVVHLLSPEQRYDPLYISNYAYLQVRDELLRLPGISDVVVWGAGEYSMRLWLDPDLIAARGLTAGEVIAAVREQNVQVAAGAVGQAPDSTAAFQVTVNTLGRLSDEEQFGDIIVR<br>TGADGQVTRLRDVARIEMGGDAYALRSLLDGEPAVALQIIQSPGANALDTAEAVRATVARLEGNFPAGLSARIAYDPTVFVRASLQTVATTLLEAILLVVVVVVLFLRSWRASLIPLMAVPVSLVGTF<br>AVMHLMGFSLNTLSLFGLVLSIGIVVDDAIVVVENVERHIENGEPPLQAARRAMDEVTGPIMAITSVLAAVFIPTAFLSGLQGEFYRQFALTIAISTILSALNSLTLSPALAGLLLRPRPAGGAVAGRFQ<br>RLLQVLGRPLRNAPEAYGNAVRKVVRVSGLALVVYGGLLGLTWVGFQAVPPGFVPMQDKYYLVGIAQLPNGASLERTDAVVRQMSRIGLDEPGVESVVAFPGLSVNGFVNVPNAAVMFFMLDP<br>FESRTSADLGALAIAGRLQARFASIPDGFLGVFPPPPVPGLGTIGGFKMQVEDRGGAGLEALARQTQVLMMKATESGQLGGLMTSFDINAPQLEVVVDRTKVKSQGVRLADVFEALQVYLGSLYIN<br>DFNRFGRTYKVTAQADAPHRMQAEAIGRLQVRNAAGAMLPLSSFVTVTPSSGPDRVIHYNGYPSADISGGALPGVSSGQAVALMERLAGEVLPEGMTFEWTDLTYQQKLAGNSALFIFPLCVLLA<br>YLILAAQYNSWLLPLAVLLIVPMCLLSAIAGVWLVGGDNNVFVQIGLVVLVGLAAKNAILIVEFARTLEAEGARALEAVVEACRLRLRPILMTSLAFIAGVVPLVMASGAGAEMRQAMGVAVFAGM<br>LGVTLFGLFLTPVFYVLVRALAARLERRAGGSLAHLEGGRP
9	MexW_Pa	PA4375 (mexW)	MAFTDPFIRRPVLASVVSLLIVLLGMQAFSKLVIREYPQMENALITVTTLYAGANAETIQGYITQPLQQSLASAEGIDYMTSVSRQNYSTISIYARIGANTDRLVTELLAKSNEVKSQLPPDAEDPVLQKE<br>AADASALMYISFYSEQMNNPQITDYLSRVIQPKLATLPGIAEAEILGNQVFAMRLWLDPVKMAAFGVTAGEINQAVQQYNFLAAAGEVKGQLVVTSVNASTDLKSPQAFAAIPVKTDGDRRVLMG<br>DVARVELGAASYDAISSFNGIPSVYIGIKGTPSANPLDVIKEVRAKMPELEEQLPPNLKVSIAYDATRFIQASIDEVVKTLGEAVLIVIVVVFLFLGAFRSVLIPVVTIPLSMIGVLFFMQAMGYSINLLTLLA<br>MVLAIGLVVDDAIVVVENIHRHIEEGKPPFEAALEGAREIAVPVVSMTITLAAVYAPIGFLTGLTGALFKEFAFTLAGAVIISGIVALTLSPMMCSRLLRHEENPSGLAHRLDLIFEGLKQRYQRALHGTLD<br>TRPVVLVFAVLVLALIPVLLMFTKKELAPEEDQGIVFLMTNSPQTANLDYLNRYTAEFEGIFRSFPEYYSAFQINGYNGVQAGIGGMLLKPWDEREKSQMELLHAVQAKLNEIPGVQIFAFNLPSLPGT<br>GEGLPFQFVLNTANDYESLLQVAQRVKQRASESGKFAFLDLDLAFDKPELVVDIDREKAAQMGVSMQDLGVALASLLGEGEINRFTIDGRSYKVIAQVERPYRDNPGWLGSYYVKSRNGQLVALST<br>LIETHERARPRQLNQFQQLNSAIISGFPIVSMGEAIETVQQIAREEAPRGFAVDYAGASRQYVQEGSALLVTFGLALAIIFLVLAAQFESFRDPLVIMVTVPLSICGALIPLFLGVSSLNIYTQVGLVTLIGL<br>ISKHGILIVEFANQLRHEQGLGRREAIEQAAAIRLRPVLMTTAAMVLGVIPLILATGAGAVSRFDIGIVIATGMSVGTLFTLFVLPCIYTLVARPDAPPGVTQAANAH
10	MuxB_Pa	PA2527 (muxB)	MNPSRPFILRPVATTLLMVAILLSGLIAYRFLPISALPEVDYPTIQVVTLYPGASPEIMTSSITAPLENQLGQIPGLNEMSSSSSGGASVITLQFSLQSNLDVAEQEVQAAINAAQSLLPNDLPNQPVFSK<br>VNPADAPILTLAVMSDGMPLPQIQDLVDTRLAQKISQISGVGLVSISGGQRPAVRVRANPTALAAAGLSLEDLRSTVTSNNLNGPKGSFDGPTRASTLDANDQLRSADAYRDLIIAYKNGSPLRIRDV<br>ASVEDDAENVRLAAWANNLPAVVLNIQRQPGANVIEVVDRIKALLPQLQSTLPGNLDVQVLTDRTTTIRASVKDVQFELALAVALVVMVTFLFLRNVYATLIPSFAVPLSLIGTFGVMYLSGFSINNLT<br>LMALTIATGFVVDDAIVMVENIARYLEQGDSPLEAALKGSKQIGFTIISLTFSLIAVLIPLLFMGDVAGRLFREFAITLAVAILISGFVSLTLTPMLSAKLLRHIDEDQQGRFARAAGRVIDGLIAQYAKALR<br>VVLRHQPLTLLVAIATLALTALLYLAMPKGFFPVQDTGVIQGVAEAPQSISFQAMSERQRALAEVVLKDPAVASLSSYIGVDGSNPTLNTGRLLINLKPHSERDVTASEVIQRLQPELDHLPGIKLYMQP<br>VQDLTIEDRVARTQYQFTLQDADPDVLAEWVPKLVARLQELPQLADVASDWQDKGLQAYLNIDRDTASRLGVKLSDIDSVLYNAFGQRLISTIFTQATQYRVVLEVAPQFQLGPQALEQLYVPSSDG<br>TQVRLSSLAKVEERHTLLAINHIAQFPSATLSFNLAKGYSLGEAVEAIRGVEASLELPLSMQGSFRGAALAFEASLSNTLLLILASVVTMYIVLGILYESFIHPVTILSTLPSAGVGALLALMLAGQEIGIVAII<br>GIILLIGIVKKNAIMMIDFALDAERNEGKPPHEAIYQACLLRFRPILMTTMAALLGALPLMLAGGAGAELRQPLGITMVGGLLLSQVLTLFTTPVIYLYFDRLARRWAAWRKQRGLDLNTEAGFDGDA<br>GR
11	MuxC_Pa	PA2526 (muxC)	MSLSTPFIRRPVATTLLTLALLLAGTLSFGLLPVAPLPNVDFPAIVVSASLPGASPETMASSVATPLERSLGRIAGISEMTSSSSLGSTTVVLVFDLEKDIDGAAREVQAAINGAMSLLPSGMPNNPSYR<br>KANPSDMPIMVLTLTSETQSRGEMYDLASTVLAPKLSQVQGVGQVSIGGSSLPAVRVDLNPDAMSQYGLSLDSVRTAIAAANSNGPKGAVEKDDKHWQVDANDQLRKAREYEPLVIHYNADNGA<br>AVRLGDVAKVSDSVEDVRNAGFSDDLPAVLLIVTRQPGANIIEATDAIHAQLPVLQELLGPQVKLNVMDDRSPSIRASLEEAELTLLISVALVILVVFLFLRNGRATLIPSLAVPVSLIGTFAVMYLCDFSL<br>NNLSLMALIIATGFVVDDAIVVVENIARRIEEGDPPIQAAITGARQVGFTVLSMTLSLVAVFIPLLLMGGLTGRLFREFAVTLSAAILVSLVVSLTLTPMLCARLLRPLKRPEGASLARRSDRFFAAFMLR<br>YRASLGWALEHSRLMVVIMLACIAMNLWLFVVVPKGFLPQQDSGRLRGYAVADQSISFQSLSAKMGEYRKILSSDPAVENVVGFIGGGRWQSSNTGSFFVTLKPIGERDPVEKVLTRLRERIAKVP<br>GAALYLNAGQDVRLGGRDSNAQYEFTLRSDDLTLLREWAPKVEAAMRKLPQLVDVNSDSQDKGVQTRLVIDRDRAATLGINVEMVDAVLNDSFGQRQVSTIFNPLNQYRVVMEVDQQYQQSPE<br>ILRQVQVIGNDGQRVPLSAFSHYEPSRAPLEVNHQGQFAATTLSFNLAPGAQIGPTREAIMQALEPLHIPVDVQTSFEGNAGAVQDTQNQMPWLILLALLAVYIVLGILYESYVHPLTILSTLPSAGVG<br>ALLALILCRSELSLIALIGIILLIGIVKKNAIMMIDFALEAERNHGLSPREAILEACMMRFRPIMMTTLAALLGALPLIFGIGGDAALRRPLGITIVGGLIGSQLLTLYTTPVVYLYLDRLRHWVNQKRGVRTD<br>GALETPL
12	CzcA_Pa	PA2520 (czcA)	MFERIIQFAIEQRWLVLLAVLGMAGVGIGSYQKLSIDAVPDITNVQVQINTAAPGYSPLEVEQRITYPVETVMAGLPGLQETRSLSRPGISQVTVIFEEGTDIYFARQQVNERLSTAREQLPEDISPTLG<br>PISTGLGEIYLWTVEAEEGATKEDGSAYTPTDLRTIQDWIIRPQLRNVKGVAEINTIGGYAKQFLIAPDPKKLAAYKLTLGDLQNAVLRNNENVGAGYIERRGEQLLIRAPGQVKDMDDIRGIIVSNVD<br>GVPIRIRDVAEVGLGKELRTGAATENGREVVLGTVFMLIGENSREVAQAVGQRLEEINRTLPKGVKAITVYDRTTLVDKAVATVKKNLVEGAALVIAVLFLFLGNIRAALITATIIPLSMLFTFTGMVG<br>NRVSANLMSLGALDFGIIVDGAVVIVENAIRRLAHAQAHHGRQLTRAERFHEVFAASREARRALVFGQIIIMVVYLPIFALTGVEGKMFHPMAFTVVTALLGAMILSVTFVPAAIALFITGKVKEEENF<br>VMRRARLAYEPALRWVLGHRALVVGGALGAILLTGLVASRMGSEFIPSLSEGDFAMQGLRVPGTSLTQSVEMQQTLERKLMGKFPEIERVFARTGTAEIASDLMPPNASDSYVMLKPQSQWPDP<br>KKSREALLEELQAAALEVPGSVYEFSQPIQLRFNELISGVRSDVAVKVFGDDMQVLNDTAEKISKVLQGIDGASEVKVEQTTGLPVLTVDIDRDKAARFGLNVGDIQDTVATALGGRNAGTLFEGDR<br>RFDIVIRLPETLRADLPALSNLLIPLPPNNLARIDFIPLSDVARLDLSPGPNQISRENGKRRIVVSANVRGRDIGSFVLEAQQKLQDGVKIPAGYWTTWGGQFEQLQSAAKRLQVVVPVALLLVFTLLFA<br>MFNNVKDGLLVFTGIPFALTGGVLALWLRGIPLSISAAVGFIALSGVAVLNGLVMISFIRNLLQEGRSLDQAVWEGAITRLRPVLMTALVASLGFVPMALATGTGAEVQRPLATVVIGGILSSTMLTL<br>LVLPVLYRWTHGRDAGPREETPAAG
13	TriC_Pa	PA0158 (triC)	MKGGFNLSDWALRHQSLVWYLMAVSLVMGVFSYLNLGREEDPSFAIKTMVIQTRWPGATVDDTLEQVTDRIEKKLEELDSLDYVKSYTRPGESTVFVYLKDTTKAGDIPDIWYQVRKKISDIQGEFP<br>QGIQGPGFNDEFGDVFGSVYAFTADGLDFRQLRDYVEKVRLDIRSVKDLGKVQMIGAQNEVIYLNFSTRKLAALGLDQRQVVQSLQAQNAVTPSGVVEAGPERISVRTSGNFRSEKDLQAVNLRV<br>NDRFYRLSDLASISRDFVDPPTSLFRYKGEPAIGLAVAMKEGGNILEFGEALNARMQEITGELPVGVGVHQVSNQAQVVKKAVGGFTRALFEAVVIVLIVSFVSLGLRAGLVVACSIPLVLAMVFVF<br>MEYTDITMQRVSLGALIIALGLLVDDAMITVEMMITRLELGDSLHDSATYAYTSTAFPMLTGTLVTAGFVPIGLNASSAGEYTFTLFAVIAVALLLSWIVAVLFAPVIAVHILPKTLKHKSEQKKGRIAE<br>RFDSLLHLAMRRRWTTIFLTALLFGVSLFLMKFVQHQFFPSSDRPELLVDLNLPQNSSIHETRAVMDRLEATLKDDEDIDHWSAYVGEGAIRFYLPLDQQLQNNFYGQLVIVTKDLEARERVAARLRD<br>RLRKDYVGISTYVQPLEMGPPVGRPIQYRVSGPQIDKVREYAMGLAGVLDGNPNIGDIVYDWNEPGKMLKIDIAQDKARQLGLSSEDVAQIMNSVVTGSAVTQVRDDIYLVNVIGRAEDSERGSLE<br>TLESLQIVTPSGTSIPLKAFAKVSYELEQPLVWRRDRKPTITVKASLRGEIQPTDLVARLAPEVKRFADGLPANYRIEVGGTVEESGKAEGPIAKVVPLMLFLMATFLMIQLQSVQKLFLVASVAPLGLIG<br>VVAALLPTGTPMGFVAILGILALIGIIIRNSVILVTQIDAFEKDGKTPWEAVLEATHHRTRPILLTAAAASLGMIPIAREVFWGPMAYAMIGGIVAATLLTLIFLPALYVAWYRIPEPGR

Table S1. **Continued.**

14	AcrB_Ec	b0462 (acrB)	MPNFFIDRPIFAWVIAIIIMLAGGLAILKLPVAQYPTIAPPAVTISASYPGADAKTVQDTVTQVIEQNMNGIDNLMYMSSNSDSTGTVQITLTFESGTDADIAQVQVQNKLQLAMPLLPQEVQQQGVSVEKSSSSFLMVVGVINTDGTMTQEDISDYVAANMKDAISRTSGVGDVQLFGSQYAMRIWMNPNELNKFQLTPVDVITAIKAQNAQVAAGQLGGTPPVKGQQLNASIIAQTRLTSTEEFGKILLKVNQDGSRVLLRDVAKIELGGENYDIIAEFNGQPASGLGIKLATGANALDTAAAIRAELAKMEPFFPSGLKIVYPYDTTPFVKISIHEVVKTLVEAIILVFLVMYLFLQNFRATLIPTIAVPVVLLGTFAVLAAFGFSINTLTMFGMVLAIGLLVDDAIVVVENVERVMAEEGLPPKEATRKSMGQIQGALVGIAMVLSAVFVPMAFFGGSTGAIYRQFSITIVSAMALSVLVALILTPALCATMLKPIAKGDHGEGKKGFFGWFNRMFEKSTHHYTDSVGGILRSTGRYLVLYLIIVVGMAYLFVRLPSSFLPDEDQGVFMTMVQLPAGATQERTQKVLNEVTHYYLTKEKNNVESVFAVNGFGFAGRGQNTGIAFVSLKDWADRPGEENKVEAITMRATRAFSQIKDAMVFAFNLPAIVELGTATGFDFELIDQAGLGHEKLTQARNQLLAEAAKHPDMLTSVRPNGLEDTPQFKIDIDQEKAQALGVSINDINTTLGAAWGGSYVNDFIDRGRVKKVYVMSEAKYRMLPDDIGDWYVRAADGQMVPFSAFSSSRWEYGSPRLERYNGLPSMEILGQAAPGKSTGEAMELMEQLASKLPTGVGYDWTGMSYQERLSGNQAPSLYAISLIVVFLCLAALYESWSIPFSVMLVVPLGVIGALLAATFRGLTNDVYFQVGLLTTIGLSAKNAILIVEFAKDLMDKEGKGLIEATLDAVRMRLRPILMTSLAFILGVMPLVISTGAGSGAQNAVGTGVMGGMVTATVLAIFFVPVFFVVVRRRFSRKNEDIEHSHTVDHH
15	AcrD_Ec	b2470 (acrD)	MANFFIDRPIFAWVLAILLCLTGTLAIFSLPVEQYPDLAPPNVRVTANYPGASAQTLENTVTQVIEQNMTGLDNLMYMSSQSSGTGQASVTLSFKAGTDPDEAVQQVQNQLQSAMRKLPQAVQNQGVTVRKTGDTNILTIAFVSTDGSMDKQDIADYVASNIQDPLSRVNGVGDIDAYGSQYSMRIWLDPAKLNSFQMTAKDVTDAIESQNAQIAVGQLGGTPSVDKQALNATINAQSLLQTPEQFRDITLRVNQDGSEVRLGDVATVEMGAEKYDYLSRFNGKPASGLGVKLASGANEMATAELVLNRLDELAQYFPHGLEYKVAYETTSFVKASIEDVVKTLLEAIALVFLVMYLFLQNFRATLIPTIAVPVVLMGTFSVLYAFGYSVNTLTMFAMVLAIGLLVDDAIVVVENVERIMSEEGLTPREATRKSMGQIQGALVGIAMVLSAVFVPMAFFGGTTGAIYRQFSITIVAAMVLSVLVAMILTPALCATLLKPLKKGEHHGQKGFFAWFNQMFNRNAERYEKGVAKILHRSLRWIVIYVLLLGGMVFLFLRLPTSFLPLEDRGMFTTSVQLPSGSTQQQTLKVVEQIEKYYFTHEKDNIMSVFATVGSGPGGNGQNVARMFIRLKDWSERDSKTGTSFAIIERATKAFNQIKEARVIASSPPAISGLGSSAGFDMELQDHAGAGHDALMAARNQLLALAAENPELTRVRHNGLDDSPQLQIDIDQRKAQALGVAIDDINDTLQTAWGSSYVNDFMDRGRVKKVYVQAAAPYRMLPDDINLWYVRNKDGGMVPFSAFATSRWETGSPRLERYNGYSAVEIVGEAAPGVSTGTAMDIMESLVKQLPNGFGLEWTAMSYQERLSGAQAPALYAISLLVVFLCLAALYESWSVPFSVMLVVPLGVIGALLATWMRGLENDVYFQVGLLTVIGLSAKNAILIVEFANEMNQKGHDLFEATLHACRQRLRPILMTSLAFIFGVLPMATSTGAGSGGQHAVGTGVMGGMISATILAIYFVPLFFVLVRRRFPLKPRPE
16	AcrF_Ec	b3266 (acrF)	MANFFIRRPIFAWVLAIILMMAGALAILQLPVAQYPTIAPPAVSVSANYPGADAQTVQDTVTQVIEQNMNGIDNLMYMSSTSDSAGSVTITLTFQSGTDPDIAQVQVQNKLQLATPLLPQEVQQQGISVEKSSSSYLMVAGFVSDNPGTTQDDISDYVASNVKDTLSRLNGVGDVQLFGAQYAMRIWLDADLLNKYKLTPVDVINQLKVQNDQIAAGQLGGTPALPGQQLNASIIAQTRFKNPEEFGKVTLRVNSDGSVVRLKDVARVELGGENYNVIARINGKPAAGLGIKLATGANALDTAKAIKAKLAELQPFFPQGMKVLYPYDTTPFVQLSIHEVVKTLFEAIMLVFLVMYLFLQNMRATLIPTIAVPVVLLGTFAILAAFGYSINTLTMFGMVLAIGLLVDDAIVVVENVERVMMEDKLPPKEATEKSMSQIQGALVGIAMVLSAVFIPMAFFGGSTGAIYRQFSITIVSAMALSVLVALILTPALCATLLKPVSAEHHENKGGFFGWFNTTFDHSVNHYTNSVGKILGSTGRYLLIYALIVAGMVVLFLRLPSSFLPEEDQGVFLTMIQLPAGATQERTQKVLDQVTDYYLKNEKANVESVFTVNGFSFSGQAQNAGMAFVSLKPWEERNGDENSAEAVIHRAKMELGKIRDGFVIPFNMPAIVELGTATGFDFELIDQAGLGHDALTQARNQLLGMAAQHPASLVSVRPNGLEDTAQFKLEVDQEKAQALGVSLSDINQTISTALGGTYVNDFIDRGRVKKLYVQADAKFRMLPEDVDKLYVRSANGEMVPFSAFTTSHWVYGSPRLERYNGLPSMEIQGEAAPGTSSGDAMALMENLASKLPAGIGYDWTGMSYQERLSGNQAPALVAISFVVVFLCLAALYESWSIPVSVMLVVPLGIVGVLLAATLFNQKNDVYFMVGLLTTIGLSAKNAILIVEFAKDLMEKEGKGVVEATLMAVRMRLRPILMTSLAFILGVLPLAISNGAGSGAQNAVGIGVMGGMVSATLLAIFFVPVFFVVIRRCFKG
17	MdtF_Ec	b3514 (mdtF)	MANYFIDRPVFAWVLAIIMMLAGGLAIMNLPVAQYPQIAPPTITVSATYPGADAQTVEDSVTQVIEQNMNGLDGLMYMSSTSDAAGNASITLTFETGTSPDIAQVQVQNKLQLAMPSLPEAVQQQGISVDKSSSNILMVAAFISDNGSLNQYDIADYVASNIKDPLSRTAGVGSVQLFGSEYAMRIWLDPQKLNKYNLVPSDVISQIKVQNNQISGGQLGGMPQAADQQLNASIIVQTRLQTPEEFGKILLKVQQDGSQVLLRDVARVELGAEDYSTVARYNGKPAAGIAIKLAAGANALDTSRAVKEELNRLSAYFPASLKTVYPYDTTPFIEISIQEVFKTLVEAIILVFLVMYLFLQNFRATIIPTIAVPVVILGTFAILSAVGFTINTLTMFGMVLAIGLLVDDAIVVVENVERVIAEDKLPPKEATHKSMGQIQRALVGIAVVLSAVFMPMAFMSGATGEIYRQFSITLISSMLLSVFVAMSLTPALCATILKAAPEGGHKPNALFARFNTLFEKSTQHYTDSTRSLLRCTGRYMVVYLLICAGMAVLFLRTPTSFLPEEDQGVFMTTAQLPSGATMVNTTKVLQQVTDYYLTKEKDNVQSVFTVGGFGFSGQGQNNGLAFISLKPWSERVGEENSVTAIIQRAMIALSSINKAVVFPFNLPAVAELGTASGFDMELLDNGNLGHEKLTQARNELLSLAAQSPNQVTGVRPNGLEDTPMFKVNVNAAKAEAMGVALSDINQTISTAFGSSYVNDFLNQGRVKKVYVQAGTPFRMLPDNINQWYVRNASGTMAPLSAYSSTEWTYGSPRLERYNGIPSMEILGEAAAGKSTGDAMKFMADLVAKLPAGVGYSWTGLSYQEALSSNQAPALYAISLVVVFLALAALYESWSIPFSVMLVVPLGVVGALLATDLRGLSNDVYFQVGLLTTIGLSAKNAILIVEFAVEMMQKEGKTPIEAIIEAARMRLRPILMTSLAFILGVLPLVISHGAGSGAQNAVGTGVMGGMFAATVLAIYFVPVFFVVVEHLFARFKKA
18	MdtB_Ec	b2075 (mdtB)	MQVLPPSSTGGPSRLFIMRPVATTLLMVAILLAGIIGYRALPVSALPEVDYPTIQVVTLYPGASPDVMTSAVTAPLERQFGQMSGLKQMSSQSSGGASVITLQFQLTLPLDVAEQEVQAAINAATNLLPSDLPNPPVYSKVNPADPPIMTLAVTSTAMPMTQVEDMVETRVAQKISQISGVGLVTLSGGQRPAVRVKLNAQAIAALGLTSETVRTAITGANVNSAKGSLDGPSRAVTLSANDQMQSAEEYRQLIIAYQNGAPIRLGDVATVEQGAENSWLGAWANKEQAIVMNVQRQPGANIISTADSIRQMLPQLTESLPKSVKVTVLSDRTTNIRASVDDTQFELMMAIALVVMIIYLFLRNIPATIIPGVAVPLSLIGTFAVMVFLDFSINNLTLMALTIATGFVVDDAIVVIENISRYIEKGEKPLAAALKGAGEIGFTIISLTFSLIAVLIPLLFMGDIVGRLFREFAITLAVAILISAVVSLTLTPMMCARMLSQESLRKQNRFSRASEKMFDRIIAAYGRGLAKVLNHPWLTLSVALSTLLLSVLLWVFIPKGFFPVQDNGIIQGTLQAPQSSSFANMAQRQRQVADVILQDPAVQSLTSFVGVDGTNPSLNSARLQINLKPLDERDDRVQKVIARLQTAVDKVPGVDLFLQPTQDLTIDTQVSRTQYQFTLQATSLDALSTWVPQLMEKLQQLPQLSDVSSDWQDKGLVAYVNVDRDSASRLGISMADVDNALYNAFGQRLISTIYTQANQYRVVLEHNTENTPGLAALDTIRLTSSDGGVVPLSSIAKIEQRFAPLSINHLDQFPVTTISFNVPDNYSLGDAVQAIMDTEKTLNLPVDITTQFQGSTLAFQSALGSTVWLIVAAVVAMYIVLGILYESFIHPITILSTLPTAGVGALLALLIAGSELDVIAIIGIILLIGIVKKNAIMMIDFALAAEREQGMSPREAIYQACLLRFRPILMTTLAALLGALPLMLSTGVGAELRRPLGIGMVGGLIVSQVLTLFTTPVIYLLFDRLALWTKSRFARHEEEA
19	MdtC_Ec	b2076 (mdtC)	MKFFALFIYRPVATILLSVAITLCGILGFRMLPVAPLPQVDFPVIIVSASLPGASPETMASSVATPLERSLGRIAGVSEMTSSSSLGSTRIILQFDFDRDINGAARDVQAAINAAQSLLPSGMPSRPTYRKANPSDAPIMILTLTSDTYSQGELYDFASTQLAPTISQIDGVGDVDVGGSSLPAVRVGLNPQALFNQGVSLDDVRTAVSNANVRKPQGALEDGTHRWQIQTNDELKTAAEYQPLIIHYNNGGAVRLGDVATVTDSVQDVRNAGMTNAKPAILLMIRKLPEANIIQTVDSIRAKLPELQETIPAAIDLQIAQDRSPTIRASLEEVEQTLIISVALVILVVFLFLRSGRATIIPAVSVPVSLIGTFAAMYLCGFSLNNLSLMALTIATGFVVDDAIVVLENIARHLEAGMKPLQAALQGTREVGFTVLSMSLSLVAVFLPLLLMGGLPGRLLREFAVTLSVAIGISLLVSLTLTPMMCGWMLKASKPREQKRLRGFGRMLVALQQGYGKSLKWVLNHTRLVGVVLLGTIALNIWLYISIPKTFFPEQDTGVLMGGIQADQSISFQAMRGKLQDFMKIIRDDPAVDNVTGFTGGSRVNSGMMFITLKPRDERSETAQQIIDRLRVKLAKEPGANLFLMAVQDIRVGGRQSNASYQYTLLSDDLAALREWEPKIRKKLATLPELADVNSDQQDNGAEMNLVYDRDTMARLGIDVQAANSLLNNAFGQRQISTIYQPMNQYKVVMEVDPRYTQDISALEKMFVINNEGKAIPLSYFAKWQPANAPLSVNHQGLSAASTISFNLPTGKSLSDASAAIDRAMTQLGVPSTVRGSFAGTAQVFQETMNSQVILIIAAIATVYIVLGILYESYVHPLTILSTLPSAGVGALLALELFNAPFSLIALIGIMLLIGIVKKNAIMMVDFALEAQRHGNLTPQEAIFQACLLRFRPIMMTTLAALFGALPLVLSGGDGSELRQPLGITIVGGLVMSQLLTLYTTPVVYLFFDRLRLRFSRKPKQTVTE
20	CusA_Ec	b0575 (cusA)	MIEWIIRRSVANRFLVLMGALFLSIWGTWTIINTPVDALPDLSDVQVIIKTSYPGQAPQIVENQVTYPLTTTMLSVPGAKTVRGFSQFGDSYVYVIFEDGTDPYWARSRVLEYLNQVQGKLPAGVSAELGPDATGVGWIYEYALVDRSGKHDLADLRSLQDWFLKYELKTIPDVAEVASVGGVVKEYQVVIDPQRLAQYGISLAEVKSALDASNQEAGGSSIELAEAEYMVRASGYLQTLDDFNHIVLKASENGVPVYLRDVAKVQIGPEMRRGIAELNGEGEVAGGVVILRSGKNAREVIAAVKDKLETLKSSLPEGVEIVTTYDRSQLIDRAIDNLSGKLLEEFIVVAVVCALFLWHVRSALVAIISLPLGLCIAFIVMHFQGLNANIMSLGGIAIAVGAMVDAAIVMIENAHKRLEEWQHQHPDATLDNKTRWQVITDASVEVGPALFISLLIITLSFIPIFTLEGQEGRLFGPLAFTKTYAMAGAALLAIVVIPILMGYWIRGKIPPESSNPLNRFLIRVYHPLLLKVLHWPKTTLLVAALSVLTVLWPLNKVGGEFLPQINEGDLLYMPSTLPGISAAEAASMLQKTDKLIMSVPEVARVFGKTGKAETATDSAPLEMVETTIQLKPQEQWRPGMTMDKIIEELDNTVRLPGLANLWVPPIRNRIDMLSTGIKSPIGIKVSGTVLADIDAMAEQIEEVARTVPGVASALAERLEGGRYINVEINREKAARYGMTVADVQLFVTSAVGGAMVGETVEGIARYPINLRYPQSWRDSPQALRQLPILTPMKQQITLADVADIKVSTGPSMLKTENARPTSWIYIDARDRDMVSVVHDLQKAIAEKVQLKPGTSVAFSGQFELLERANHKLKLMVPMTLMIIFVLLYLAFRRVGEALLIISSVPFALVGGIWLLWWMGFHLSVATGTGFIALAGVAAEFGVVMLMYLRHAIEAVPSLNNPQTFSEQKLDEALYHGAVLRVRPKAMTVAVIIAGLLPILWGTGAGSEVMSRIAAPMIGGMITAPLLSLFIIPAAYKLMWLHRHRVRK
21	AcrB_Sa	STY0519 (acrB)	MPNFFIDRPIFAWVIAIIIMLAGGLAILKLPVAQYPTIAPPAVTISATYPGADAKTVQDTVTQVIEQNMNGIDNLMYMSSNSDSTGTVQITLTFESGTDADIAQVQVQNKLQLAMPLLPQEVQQQGVSVEKSSSSFLMVVGVINTDGTMTQEDISDYVAANMKDPISRTSGVGDVQLFGSQYAMRIWMNPTELTKYQLTPVDVINAIKAQNAQVAAGQLGGTPPVKGQQLNASIIAQTRLTSTDEFGKILLKVNQDGSQVRLRDVAKIELGGENYDVIAKFNGQPASGLGIKLATGANALDTATAIRAELKKMEPFFPPGMKIVYPYDTTPFVKISIHEVVKTLVEAIILVFLVMYLFLQNFRATLIPTIAVPVVLLGTFAVLAAFGFSINTLTMFGMVLAIGLLVDDAIVVVENVERVMTEEGLPPKEATRKSMGQIQGALVGIAMVLSAVFIPMAFFGGSTGAIYRQFSITIVSAMALSVLVALILTPALCATMLKPVAKGDHGEGKKGFFGWFNRLFDKSTHHYTDSVGNILRSTGRYLLLYLIIVVGMAYLFVRLPSSFLPDEDQGVFLTMVQLPAGATQERTQKVLDEVTDYYLNKEKANVESVFAVNGFGFAGRGQNTGIAFVSLKDWADRPGEKNKVEAITQRATAAFSQIKDAMVFAFNLPAIVELGTATGFDFELIDQAGLGHEKLTQARNQLFGEVAKYPDLLVGVRPNGLEDTPQFKIDIDQEKAQALGVSISDINTTLGAAWGGSYVNDFIDRGRVKKVYVMSEAKYRMLPDDINDWYVRGSDGQMVPFSAFSSSRWEYGSPRLERYNGLPSMEILGQAAPGKSTGEAMAMMEELASKLPSGIGYDWTGMSYQERLSGNQAPALYAISLIVVFLCLAALYESWSIPFSVMLVVPLGVIGALLAATFRGLTNDVYFQVGLLTTIGLSAKNAILIVEFAKDLMDKEGKGLVEAMLEAVRMRLRPILMTSLAFMLGVMPLVISSGAGSGAQNAVGTGVLGGMVTATVLAIFFVPVFFVVVRRRFSRKSEDIEHSHSTEHR
22	AcrD_Sa	STY2719 (acrD)	MANFFIDRPIFAWVLAILLCLTGALAIFSLPVEQYPDLAPPNVRITANYPGASAQTLENTVTQVIEQNMTGLDNLMYMSSQSSGTGQATITLSFIAGTDPDEAVQQVQNQLQSAMRKLPQAVQDQGVTVRKTGDTNILTIAFVSTDGSMDKQDIADYVASNIQDPLSRVNGVGDIDAYGSQYSMRIWLDPAKLNSFQMTTKDVTDAIESQNAQIAVGQLGGTPSVDKQALNATINAQSLLQTPQQFRDITLRVNQDGSEVKLGDVATVELGAEKYDYLSRFNGNPASGLGVKLASGANEMATAKLVLDRLNELAQYFPHGLEYKIAYETTSFVKASIIDVVKTLLEAIALVFLVMYLFLQNFRATLIPTIAVPVVLMGTFSVLYAFGYSINTLTMFAMVLAIGLLVDDAIVVVENVERIMSEEGLTPREATRKSMGQIQGALVGIAMVLSAVFVPMAFFGGTTGAIYRQFSITIVSAMVLSVLVAMILTPALCATLLKPLHKGEQHGQRGFFGWFNRTFNRNAERYEKGVAKILHRSLRWILIYVLLLGGMVFLFLRLPTSFLPQEDRGMFTTSIQLPSGSTQQQTLKVVEKVENYYFTHEKNNIMSVFSTVGSGPGGNGQNVARMFVRLKDWDARDPTTGSSFAIIERATKAFNQIKEARVFASSPPAISGLGSSAGFDMELQDHAGAGHDALMAARDQLIELAGKNSSLTRVRHNGLDDSPQLQIDIDQRKAQALGVSIDDINDTLQTAWGSSYVNDFMDRGRVKKVYVQAAAKYRMLPDDINLWYVRNKDGGMVPFSAFATSRWETGSPRLERYNGYSAVEIVGEAAPGVSTGTAMDVMESLVHQLPGGFGLEWTAMSYQERLSGAQAPALYAISLLVVFLCLAALYESWSVPFSVMLVVPLGVIGALLATWMRGLENDVYFQVGLLTVIGLSAKNAILIVEFANEMNQKGHALLDATLYASRQRLRPILMTSLAFIFGVLPMATSTGAGSGSQHAVGTGVMGGMISATILAIFFVPLFFVLIRRRFPLKPRPE
23	AcrF_Sa	STM3391 (acrF)	MANFFIRRPIFAWVLAIILMMAGALAIMQLPVAQYPTIAPPAVSISATYPGADAQTVQDTVTQVIEQNMNGIDNLMYMSSTSDSAGSVTITLTFQSGTDPDIAQVQVQNKLQLATPLLPQEVQQQGISVEKSSSSFLMVAGFVSDNPNTTQDDISDYVASNIKDSISRLNGVGDVQLFGAQYAMRIWLDANLLNKYQLTPVDVINQLKVQNDQIAAGQLGGTPALPGQQLNASIIAQTRLKDPEEFGKVTLRVNTDGSVVHLKDVARIELGGENYNVVARINGKPASGLGIKLATGANALDTATAIKVKLAELQPFFPQGMKVVYPYDTTPFVKISIHEVVKTLFEAIILVFLVMYLFLQNIRATLIPTIAVPVVLLGTFAVLAAFGYSINTLTMFGMVLAIGLLVDDAIVVVENVERVMMEDNLSPREATEKSMSQIQGALVGIAMVLSAVFIPMAFFGGSTGAIYRQFSITIVSAMALSVLVALILTPALCATLLKPVSAEHHEKKSGFFGWFNTRFDHSVNHYTNSVSGIVRNTGRYLIIYLLIVVGMAVLFLRLPTSFLPEEDQGVFLTMIQLPSGATQERTQKVLDQVTHYYLNNEKANVESVFTVNGFSFSGQGQNSGMAFVSLKPWEERNGEENSVEAVIARATRAFSQIRDGLVFPFNMPAIVELGTATGFDFELIDQGGLGHDALTKARNQLLGMVAKHPDLLVRVRPNGLEDTPQFKLDVDQEKAQALGVSLSDINETISAALGGYYVNDFIDRGRVKKVYVQADAQFRMLPGDINNLYVRSANGEMVPFSTFSSARWIYGSPRLERYNGMPSMELLGEAAPGRSTGEAMSLMENLASQLPNGIGYDWTGMSYQERLSGNQAPALYAISLIVVFLCLAALYESWSIPFSVMLVVPLGVVGALLAASLRGLNNDVYFQVGLLTTIGLSAKNAILIVEFAKDLMEKEGRGLIEATLEASRMRLRPILMTSLAFILGVMPLVISRGAGSGAQNAVGTGVMGGMLTATLLAIFFVPVFFVVVKRRFNRHHD
24	MdsB_Sa	WP_000673160.1	MKFTHFFIARPIFAIVLSLLMLLAGAIAFLKLPLSEYPAVTPPTVQVSASYPGANPQVIADTVAAPLEQVINGVDGMLYMNTQMAIDGRMVISIAFEQGTDPDMAQIQVQNRVSRALPRLPEEVQRIGVVTEKTSPDMLMVVHLVSPQKRYDSLYLSNFAIRQVRDELARLPGVGDVLVWGAGEYAMRVWLDPAKIANRGLTASDIVTALREQNVQVAAGSVGQQPEASAAFQMTVNTLGRLTSEEQFGEIVVKIGADGEVTRLRDVARVTLGADAYTLRSLLNGEAAPALQIIQSPGANAIDVSNAIRGKMDELQQNFPQDIEYRIAYDPTVFVRASLQSVAITLLEALVLVVLVVVMFLQTWRASIIPLVAVPVSLVGTFALMHLFGFSLNTLSLFGLVLSIGIVVDDAIVVVENVERHISQGKSPGEAAKKAMDEVTGPILSITSVLTAVFIPSAFLAGLQGEFYRQFALTIAISTILSAINSLTLSPALAAILLRPHHDTAKADWLTRLMGTVTGGFFHRFNRFFDSASNRYVSAVRRAVRGSVIVMVLYAGFVGLTWLGFHQVPNGFVPAQDKYYLVGIAQLPSGASLDRTEAVVKQMSAIALAEPGVESVVVFPGLSVNGPVNVPNSALMFAMLKPFDEREDPSLSANAIAGKLMHKFSHIPDGFIGIFPPPPVPGLGATGGFKLQIEDRAELGFEAMTKVQSEIMSKAMQTPELANMLASFQTNAPQLQVDIDRVKAKSMGVSLTDIFETLQINLGSLYVNDFNRFGRAWRVMAQADAPFRMQQEDIGLLKVRNAKGEMIPLSAFVTIMRQSGPDRIIHYNGFPSVDISGGPAPGFSSGQATDAIEKIVRETLPEGMVFEWTDLVYQEKQAGNSALAIFALAVLLAFLILAAQYNSWSLPFAVLLIAPMSLLSAIVGVWVSGGDNNIFTQIGFVVLVGLAAKNAILIVEFARAKEHDGADPLTAVLEASRLRLRPILMTSFAFIAGVVPLVLATGAGAEMRHAMGIAVFAGMLGVTLFGLLLTPVFYVVVRRMALKRENRVDSHDQQA
25	MdtB_Sa	WP_001197800.1	MQVLPPGSTGGPSRLFILRPVATTLLMAAILLAGIIGYRFLPVAALPEVDYPTIQVVTLYPGASPDVMTSAVTAPLERQFGQMSGLKQMSSQSSGGASVVTLQFQLTLPLDVAEQEVQAAINAATNLLPSDLPNPPIYSKVNPADPPIMTLAVTSNAMPMTQVEDMVETRVAQKISQVSGVGLVTLAGGQRPAVRVKLNAQAVAALGLTSETVRTAITGANVNSAKGSLDGPERAVTLSANDQMQSADEYRRLIIAYQNGAPVRLGDVATVEQGAENSWLGAWANQAPAIVMNVQRQPGANIIATADSIRQMLPQLTESLPKSVKVTVLSDRTTNIRASVRDTQFELMLAIALVVMIIYLFLRNIPATIIPGVAVPLSLIGTFAVMVFLDFSINNLTLMALTIATGFVVDDAIVVIENISRYIEKGEKPLAAALKGAGEIGFTIISLTFSLIAVLIPLLFMGDIVGRLFREFAVTLAVAILISAVVSLTLTPMMCARMLSQQSLRKQNRFSRACERMFDRVIASYGRGLAKVLNHPWLTLSVAFATLLLSVMLWIVIPKGFFPVQDNGIIQGTLQAPQSSSYASMAQRQRQVAERILQDPAVQSLTTFVGVDGANPTLNSARLQINLKPLDARDDRVQQVISRLQTAVATIPGVALYLQPTQDLTIDTQVSRTQYQFTLQATTLDALSHWVPKLQNALQSLPQLSEVSSDWQDRGLAAWVNVDRDSASRLGISMADVDNALYNAFGQRLISTIYTQANQYRVVLEHNTASTPGLAALETIRLTSRDGGTVPLSEIARIEQRFAPLSINHLDQFPVTTFSFNVPEGYSLGDAVQAILDTEKTLALPADITTQFQGSTLAFQAALGSTVWLIVAAVVAMYIVLGVLYESFIHPITILSTLPTAGVGALLALIIAGSELDIIAIIGIILLIGIVKKNAIMMIDFALAAEREQGMSPRDAIFQACLLRFRPILMTTLAALLGALPLMLSTGVGAELRRPLGIAMVGGLLVSQVLTLFTTPVIYLLFDRLSLYVKSRFPRHKEEA
26	MdtC_Sa	WP_001210106.1	MRFFALFIYRPVATILIAAAITLCGILGFRLLPVAPLPQVDFPVIMVSASLPGASPETMASSVATPLERSLGRIAGVNEMTSSSSLGSTRIILEFNFDRDINGAARDVQAAINAAQSLLPSGMPSRPTYRKANPSDAPIMILTLTSESWSQGKLYDFASTQLAQTIAQIDGVGDVDVGGSSLPAVRVGLNPQALFNQGVSLDEVREAIDSANVRRPQGAIEDSVHRWQIQTNDELKTAAEYQPLIIHYNNGAAVRLGDIASVIDSVQDVRNAGMTNAKPAILLMIRKLPEANIIQTVDGIRAKLPELRAMIPAAIDLQIAQDRSPTIRASLQEVEETLAISVALVILVVFLFLRSGRATLIPAVAVPVSLIGTFAAMYLCGFSLNNLSLMALTIATGFVVDDAIVVLENIARHLEAGMKPLQAALQGTREVGFTVISMSLSLVAVFLPLLLMGGLPGRLLREFAVTLSVAIGISLVVSLTLTPMMCGWMLKSSKPRTQPRKRGVGRLLVALQQGYGTSLKWVLNHTRLVGVVFLGTVALNIWLYIAIPKTFFPEQDTGVLMGGIQADQSISFQAMRGKLQDFMKIIRDDPAVNNVTGFTGGSRVNSGMMFITLKPRGERKETAQQIIDRLRVKLAKEPGARLFLMAVQDIRVGGRQANASYQYTLLSDSLAALREWEPKIRKALSALPQLADVNSDQQDNGAEMNLIYDRDTMSRLGIDVQAANSLLNNAFGQRQISTIYQPMNQYKVVMEVDPRYSQDISALEKMFVINRDGKAIPLSYFAQWRPANAPLSVNHQGLSAASTIAFNLPTGTSLSQATEAINRTMTQLGVPPTVRGSFSGTAQVFQQTMNSQLILIVAAIATVYIVLGILYESYVHPLTILSTLPSAGVGALLALELFNAPFSLIALIGIMLLIGIVKKNAIMMVDFALEAQRSGGLTPEQAIFQACLLRFRPIMMTTLAALFGALPLVLSGGDGSELRQPLGITIVGGLVMSQLLTLYTTPVVYLFFDRLRLRFSRKNSKPVVEI

All 26 RND sequences used for phylogenetic analysis are shown for *P. aeruginosa, E. coli* and *Salmonella*.

Table S2. **Relationship between the OMP and PAP–RND genes' operons.**

	#	Tripratite name	Protein (OMP) name	PAP-RND operon	OMP operon	OMP – PAP/RND operon relationship
P. aeruginosa	1	MexAB-OprM	OprM_Pa	*mexRmexABoprM*	*mexRmexABoprM*	OMP together with PAP-RND in operon
	2	MexCD-OprJ	OprJ_Pa	*mexCDoprJ*	*mexCDoprJ*	OMP together with PAP-RND in operon
	3	MexEF-OprN	OprN_Pa	*mexSTmexEFOprN*	*mexSTmexEFOprN*	OMP together with PAP-RND in operon
	4	MexXY-OprM	OprM_Pa	*mexZXY*	*mexRmexABoprM*	OMP from different PAP-RND-OMP operon
	5	MexMN-OprM	OprM_Pa	*mexMN*	*mexRmexABoprM*	OMP from different PAP-RND-OMP operon
	6	MexJK-OprM	OprM_Pa	*mexLJK*	*mexRmexABoprM*	OMP from different PAP-RND-OMP operon
	7	MexJK-OprH	OprH_Pa	*mexLJK*	*oprHphoPQ*	OMP from different operon
	8	MexHI-OpmD	OpmD_Pa	*mexHIopmD*	*mexHIopmD*	OMP together with PAP-RND in operon
	9	MexPQ-OpmE	OpmE_Pa	*mexPQopmE*	*mexPQopmE*	OMP together with PAP-RND in operon
	10	MexVW-OprM	OprM_Pa	*mexVW*	*mexRmexABoprM*	OMP from different PAP-RND-OMP operon
	11	MuxABC-OpmB	OpmB_Pa	*muxABCopmB*	*muxABCopmB*	OMP together with PAP-RND in operon
	12	CzcBAC	CzcC_Pa	*czcRSczcCBA*	*czcRSczcCBA*	OMP together with PAP-RND in operon
	13	TriABC-OprH	OprH_Pa	*triABC*	*oprHphoPQ*	OMP from different PAP-RND-OMP operon
E. coli	14	AcrAB-TolC	TolC_Ec	*acrAB*	*tolC-ygiABC*	OMP from different operon
	15	AcrAD-TolC	TolC_Ec	*acrD*	*tolC-ygiABC*	OMP from different operon
	16	AcrEF-TolC	TolC_Ec	*acrEF*	*tolC-ygiABC*	OMP from different operon
	17	MdtEF-TolC	TolC_Ec	*gadE-mdtEF*	*tolC-ygiABC*	OMP from different operon
	18	MdtABC-TolC	TolC_Ec	*mdtABCD-baeSR*	*tolC-ygiABC*	OMP from different operon
Salmonella	19	AcrAB-TolC	TolC_Sa	*acrAB*	*tolC*	OMP from different operon
	20	AcrAD-TolC	TolC_Sa	*acrD*	*tolC*	OMP from different operon
	21	AcrEF-TolC	TolC_Sa	*acrEF*	*tolC*	OMP from different operon
	22	MdsAB(C/TolC)	TolC_Sa	*MdsABC*	*MdsABC / tolC*	OMP together with PAP-RND in operon / OMP from different operon
	23	MdtABC-TolC	TolC_Sa	*mdtABCD-baeSR*	*tolC*	OMP from different operon

All 23 PAP–RND–OMP tripartite efflux pumps systems are shown for *P. aeruginosa, E. coli* and *Salmonella*. For *P. aeruginosa,* most OMP genes are in the same operon as their PAP–RND counterparts, while for *E. coli* and *Salmonella*, the OMP (TolC) genes are generally expressed from a seperate operon.
